# Targeted therapies in bladder cancer: signaling pathways, applications, and challenges

**DOI:** 10.1002/mco2.455

**Published:** 2023-12-15

**Authors:** Mei Peng, Xuetong Chu, Yan Peng, Duo Li, Zhirong Zhang, Weifan Wang, Xiaochen Zhou, Di Xiao, Xiaoping Yang

**Affiliations:** ^1^ Department of Pharmacy Xiangya Hospital Central South University Changsha Hunan China; ^2^ Key Laboratory of Study and Discovery of Small Targeted Molecules of Hunan Province The Research Center of Reproduction and Translational Medicine of Hunan Province Key Laboratory of Chemical Biology & Traditional Chinese Medicine Research of Ministry of Education Department of Pharmacy School of Medicine Hunan Normal University Changsha Hunan China

**Keywords:** ADCs, bladder cancer, CSCs, ICIs, metabolism, TKIs

## Abstract

Bladder cancer (BC) is one of the most prevalent malignancies in men. Understanding molecular characteristics via studying signaling pathways has made tremendous breakthroughs in BC therapies. Thus, targeted therapies including immune checkpoint inhibitors (ICIs), antibody–drug conjugates (ADCs), and tyrosine kinase inhibitor (TKI) have markedly improved advanced BC outcomes over the last few years. However, the considerable patients still progress after a period of treatment with current therapeutic regimens. Therefore, it is crucial to guide future drug development to improve BC survival, based on the molecular characteristics of BC and clinical outcomes of existing drugs. In this perspective, we summarize the applications and benefits of these targeted drugs and highlight our understanding of mechanisms of low response rates and immune escape of ICIs, ADCs toxicity, and TKI resistance. We also discuss potential solutions to these problems. In addition, we underscore the future drug development of targeting metabolic reprogramming and cancer stem cells (CSCs) with a deep understanding of their signaling pathways features. We expect that finding biomarkers, developing novo drugs and designing clinical trials with precisely selected patients and rationalized drugs will dramatically improve the quality of life and survival of patients with advanced BC.

## Graphical abstract

Advances in understanding molecular characteristics of bladder cancer have prompted its therapeutic breakthroughs. Newly approved targeted drugs including immune checkpoint inhibitors (ICIs), antibody–drug conjugates (ADCs), and tyrosine kinase inhibitor (TKI) have greatly improved the survival outcomes of advanced patients. Furthermore, targeting metabolism reprogramming and cancer stem cells (CSCs) are full of revolutionary potential.

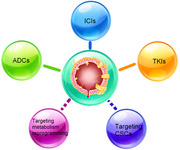

## 1 INTRODUCTION

Bladder cancer (BC) is the most common malignancy of the urinary system, accounting for 90−95% of urothelial carcinomas (UC), including tumors of the urinary bladder, upper urinary tract, and proximal urethra.^1^ Statistical data have revealed that by 2022, there will be approximately 81,180 new cases of BC in the United States of America.^2^ Tumors are classified using the tumor–node–metastasis system, which measures the depth of bladder wall invasion (Tis‐T4)^3^ or the World Health Organization International Society of Urological Pathology criteria, which classifies BC into high‐ or low‐grade disease based on histomorphological features.^4^ Tumors confined to the urothelium (stage Ta) and lamina propria (stage T1) are categorized as nonmuscle‐invasive bladder cancer (NMIBC), whereas those invading the muscle (stage T2) or beyond (stages T3 and T4) are categorized as muscle‐invasive bladder cancer (MIBC). Low‐grade NMIBC recurs frequently but rarely progresses to muscle invasion, whereas high‐grade MIBC easily progresses to metastasis, implying distinct characteristics between these two classes of tumors.

The genomic landscape of BC has been explored using novel technologies, such as next‐generation sequencing. NMIBC commonly have a near‐diploid karyotype and few genomic rearrangements. In contrast, MIBC are aneuploid with many alterations, including chromothripsis.^5^ Inactivating mutations in ataxia‐telangiectasia have been reported in MIBC and play an important role in DNA damage repair. Stromal antigen 2 (STAG2) is frequently mutated in NMIBC. It encodes a component of the cohesion complex involved in chromatid segregation.^6^ In addition to point mutations, the somatic mutation frequency in MIBC is as high as 300 exonic mutations per sample, with mean and median rates of 7.7 and 5.5 per megabase, respectively.^7^ Chromosomal alterations, such as chromosome 9 deletion, are common in, both, NMIBC and MIBC. The candidate tumor suppressor genes affected are cyclin‐dependent kinase inhibitor 2A, patched 1, and tuberous sclerosis 1.^8^ Approximately 80% of fibroblast growth factor receptor (FGFR) 3‐activating mutations and 25% of phosphatidylinositol‐4,5‐bisphosphate 3‐kinase catalytic subunit alpha (PIK3CA) mutations manifest in patients with NMIBC. TP53‐ and RB1‐inactivating mutations are common in MIBC. In addition, growth factor receptors, such as epidermal growth factor receptor (EGFR), human epidermal growth factor receptor 2 (ERBB2), and ERBB3, are upregulated in MIBC. Genomic changes in BC lead to alterations in signaling pathways. The phosphatidylinositol 3‐kinase/protein kinase B (PI3K/AKT) and mitogen‐activated protein kinase (MAPK) signal transduction pathways are frequently activated in NMIBC due to FGFR3, PI3K, and RAS oncogenic mutations. Cell cycle and checkpoint control are disturbed by TP53 inactivation and RB1 mutations and deletions in MIBC.^9^

The differences in molecular characteristics between NMIBC and MIBC ultimately affect the clinical treatment of BC. For approximately 70% of patients with NMIBC at first diagnosis, the standard treatment is transurethral resection of bladder tumor (TURBT), followed by intravesical chemotherapy or immune inhibitor. However, approximately 31−78% of NMIBC cases relapse within 5 years.^10^ Moreover, 30% of patients with BC are diagnosed with MIBC, which is associated with poor prognosis and a high mortality rate (approximately 50%).^11^ For a long time, radical cystectomy and systematic chemotherapy have been the conventional strategies for treating BC.^12^ Recent advances in understanding the molecular features of BC have updated treatment paradigms. The high mutational burden of BC renders it susceptible to immunotherapy, particularly to checkpoint inhibitors (CPIs). FGFR, a receptor tyrosine kinase, is a BC target, and erdafitinib, an oral pan‐FGFR inhibitor, has been approved for the treatment of BC. Antibody–drug conjugates (ADCs) exploit highly expressed tumor proteins as targets for drug delivery. Enfortumab vedotin (EV), which links the microtubule‐disrupting molecule monomethyl auristatin E with an antinectin‐4 antibody, and sacituzumab govitecan (SG), which uses an antitrophoblast cell surface marker 2 antibody linked to a topoisomerase inhibitor, have been approved for the treatment of BC.^13^ These newly approved drugs have improved the management of BC and have significantly benefited patients with BC.

In addition, other targeted therapies are also being explored. Metabolic alterations are hallmarks of cancer and regulate tumor progression and metastasis.^14^ Targeting metabolic reprogramming, such as aerobic glycolysis, has attracted considerable attention. Researchers have analyzed the differences between cancer and normal cells to identify suitable targets for BC therapeutics.^15^ Changes in metabolic signaling pathways have been observed in BC, and some potential metabolic targets have been validated.^16^ Cancer stem cells (CSCs) are undifferentiated cells exhibiting stem cell‐like properties. They are crucial for tumorigenesis, cancer progression, and drug resistance.^17^ CSCs are partly responsible for the unsuccessful clinical treatment of cancer. In 2009, CSCs were first identified in BC.^18^ Advances in CSC biology have made them ideal targets for BC therapy.^19^

This review aims to update information on targeted therapies for BC, including immunotherapy, ADCs, tyrosine kinase inhibitors (TKIs), metabolism, and CSCs. In addition, the review elucidates the clinical efficacy of the approved drugs, highlights the challenges in their clinical use, and explores potential solutions. The review also summarizes the latest research progress on metabolism and CSC in BC, focusing on alternating signaling pathways and investigating potential targets for targeted drug development.

## 2 IMMUNOTHERAPY IN BC

Immunotherapy induces a strong antitumor immune response and has great potential for cancer treatment. BC is treated using various immunotherapeutic approaches. Bacillus Calmette–Guérin (BCG) intravesical instillation is used in the early stages of cancer, whereas five recently approved immune checkpoint inhibitors (ICIs) are used at advanced stages.^20^ Here, we have provided updated information regarding BC immunotherapy from the perspective of BCG and ICIs (Table 1). We have interpreted the principles of BCG and ICIs, summarized their clinical efficacy, and highlighted the challenges and strategies of current immunotherapies for BC.

**TABLE 1:** Approved immunotherapies in bladder cancer until 2022.

Drugs	Target	Line of therapy	Indication in BC	Approval time	Note
BCG	/	/	Intermediate‐risk NMIBC	1990	/
Pembrolizumab	PD‐1	/	BCG‐unresponsive, high‐risk NMIBC	2020	/
Pembrolizumab	PD‐1	First‐line	Platinum‐unfit metastatic MIBC	2017	/
Atezolizumab	PD‐L1	First‐line	Platinum‐unfit metastatic MIBC	2017	/
Pembrolizumab	PD‐1	Second‐line	Platinum‐failure metastatic MIBC	2017	/
Atezolizumab	PD‐L1	Second‐line	Platinum‐failure metastatic MIBC	2016	Voluntarily withdrawn in March 2021
Nivolumab	PD‐1	Second‐line	Platinum‐failure metastatic MIBC	2017	/
Avelumab	PD‐L1	Second‐line	Platinum‐failure metastatic MIBC	2017	/
Durvalumab	PD‐L1	Second‐line	Platinum‐failure metastatic MIBC	2017	Voluntarily withdrawn in February 2021

Abbreviations: BC, bladder cancer; NMIBC, nonmuscle invasive bladder cancer.

### 2.1 Intravesical instillation with BCG as a gold standard for high‐risk NMIBC

The BCG vaccine has been used for a long time to prevent tuberculosis.^21^ In the early 20th century, William B. Coley pioneered the immunotherapeutic concept of treating patients with cancer using microbial products.^22^, ^23^ The antitumor activity of BCG has been investigated against several cancers. Fortunately, BCG exerts anticancer effects in BC. Intravesical BCG instillation for the treatment of NMIBC was first reported in 1976^24^ and approved in 1990, becoming the first immunotherapy.^25^ Currently, BCG immunotherapy is the gold standard adjuvant treatment for NMIBC and is also recommended for treating intermediate‐risk NMIBC.^10^ The European Association of Urology guidelines for NMIBC, published in 2019, have prescribed an intravesical BCG treatment schedule comprising a 6‐week induction course with weekly instillations, followed by at least three BCG maintenance courses, each consisting of weekly instillation for 3 weeks at 3, 6, and 12 months after the start of BCG induction.^10^

#### 2.1.1 Effect of BCG on immune signaling pathways on BC

**FIGURE 1:**
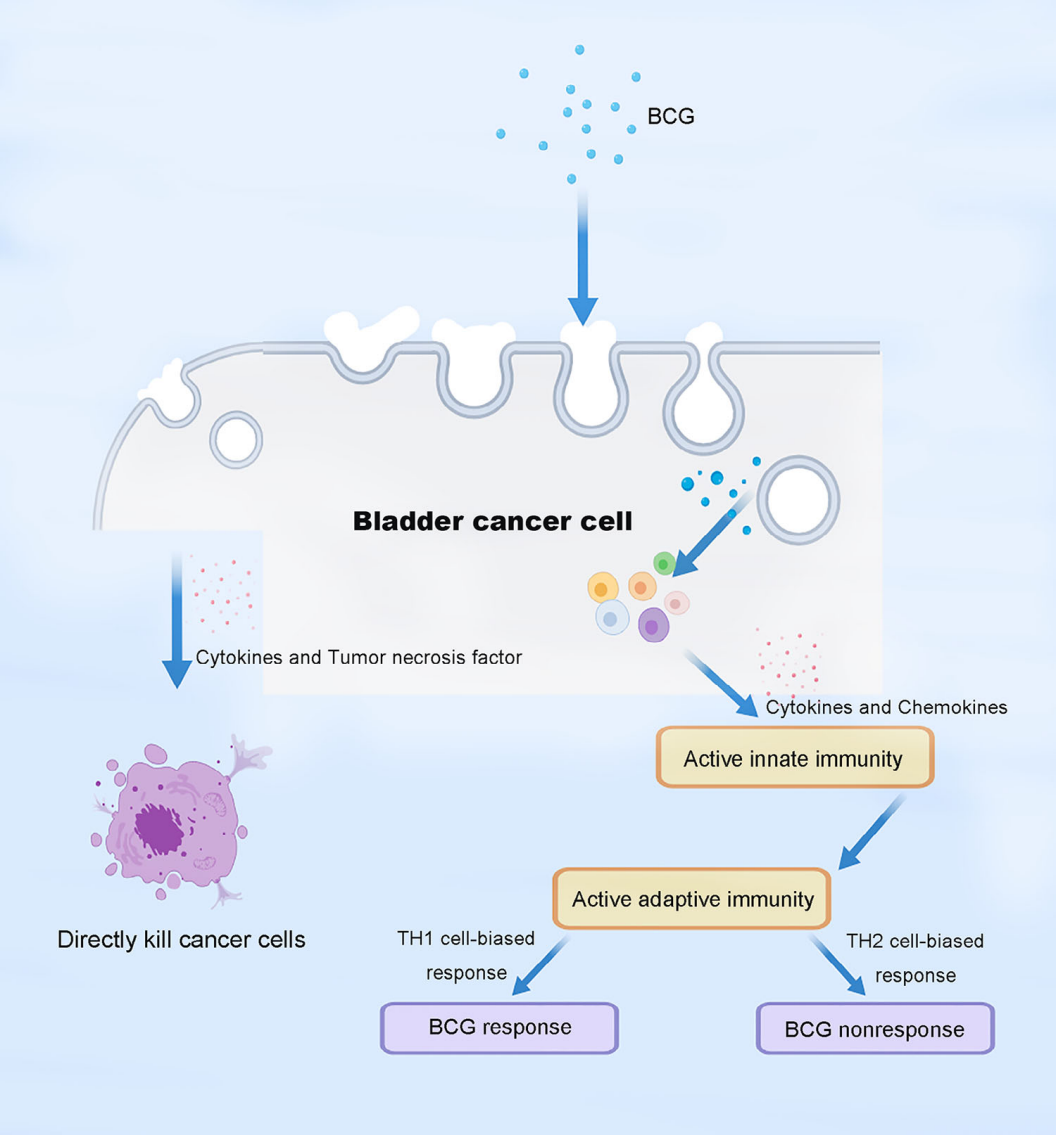
Effect of BCG on immune signaling pathways on bladder cancer. First, BCG intravesical instillation is internalized by bladder cancer cells. Then, it kills bladder cancer cells directly through inducing cytokines. Furthermore, it recruits immune cells such as T lymphocytes, macrophages, and neutrophils and activates innate immune response. A robust innate response supports a strong T helper 1 (TH1) cell‐biased adaptive immune response while a T helper 2 (TH2) cell‐biased T cell response is correlated with BCG nonresponse. BCG, Bacille Calmette‐Guérin.

BCG exerts its antitumor effects on BC by interacting with cancer cells, activating the initial immune response, and inducting adaptive immunity (Figure 1). After intravesical instillation, BCG attaches to urothelial cells through fibronectin and integrin α5β1. It is then internalized by macrophages and neutrophils via phagocytosis. BC cells internalize BCG via micropinocytosis.^26^ BCG can directly kill BC cells by inducing the release of cytokines such as interleukin (IL)‐6, IL‐8, and tumor necrosis factor (TNF).^27^ Moreover, intravesical BCG instillation activates urothelial and antigen‐presenting cells (APCs), producing cytokines and chemokines that recruit immune cells.^28^ Lymphocytes are a component of the inflammatory infiltrate in the bladders of patients treated with BCG, and CD4^+^ T cells infiltrate the bladder mucosa months after intravesical instillation of BCG.^29^ Macrophages and cytokines are found in the bladder walls and urine of patients treated with BCG. Macrophage cytotoxicity against BC cells can be stimulated by IL‐2, IL‐12, and TNF, indirectly linking macrophages to BCG.^30^ Neutrophils may directly kill cancer cells as they are phagocytic.^31^ Natural killer (NK) cells are a part of the innate immune system and kill tumor cells in an antigen‐independent manner. However, the role of NK cells in BCG‐induced antitumor activity is unclear.

BCG treatment also induces adaptive immunity with crucial roles of T cells. Dendritic cells (DCs) and urothelial cells ingest and present BCG antigens via major histocompatibility complex (MHC) class II molecules, which further interact with CD4^+^ T cell receptors, leading to the activation and primarily differentiate to T helper 1 (TH1) cell immune responses.^28^ Cytotoxic CD8^+^ T lymphocytes recognize tumor cells through antigen presentation via MHC class I molecules. Their activation is facilitated by a TH1 cell cytokine environment and is mediated by interferon (IFN)γ. The balance between TH1 and TH2 cell responses is consequential, as a TH1 cell‐biased response, characterized by the production of IL‐2, IL‐12, IFNγ, TNF, and TNFβ, is associated with successful BCG immunotherapy, whereas a TH2 cell response, characterized by the production of IL‐4, IL‐5, IL‐6, and IL‐10, correlates with BCG nonresponsiveness.^32^, ^33^

#### 2.1.2 Clinical application of BCG

Approximately half of the patients with NMIBC who have undergone TURBT alone experience tumor recurrence or progression and even metastasis.^34^ Substantial evidence supports that BCG reduces the risk of tumor progression, especially with maintenance intravesical BCG treatment. Yamamoto et al.^35^ conducted a prospective randomized study to determine whether intravesical BCG prevents NMIBC recurrence. They observed a significantly lower recurrence rate during follow‐up in patients with sustained intravesical instillation of 80 mg BCG than in those treated with TURBT alone. In addition, side effects such as cystitis and fever were controllable, and treatment was discontinued in only two patients. Thus, intravesical BCG exerts a prophylactic effect against NMIBC recurrence after TURBT.

Pagano et al.^36^ evaluated the efficacy and toxicity of sustained low‐dose (75 mg) BCG in NMIBC. The patients were divided into the following three groups: prophylaxis (*n* = 70), control (*n* = 63), and therapy (*n* = 56). The response rates in the three groups were 74, 17, and 57%, respectively. Moreover, 4, 17, and 12.5% of patients experienced tumor progression, with toxicity, cystitis, and fever as the common symptoms. This study indicated that a sustained low dose (75 mg) of intravesical BCG effectively prevented NMIBC. The benefits of intravesical BCG have also been confirmed in other studies.^37^, ^38^

#### 2.1.3 The challenges of BCG treatment

Despite the success of BCG in treating NMIBC, approximately 50% of high‐risk patients with NMIBC eventually experience tumor recurrence. In addition, up to 30% of these patients develop MIBC.^39^ Therefore, it is important to identify potential signaling molecules that could predict BCG response or be associated with BCG resistance and then find potential solutions.

Age, sex, genetic polymorphisms, high mutational burden, and microbial ecosystems are associated with the response to intravesical BCG.^40^ Recently, immunological parameters for predicting BCG responses have been determined based on retrospective studies. For instance, the systemic immune inflammation index, and the levels of tumor‐infiltrating immune cells, urinary IL‐10, serum cytotoxic T‐lymphocyte‐associated protein 4 (CTLA‐4), and transcription factors can predict BCG response.^41^, ^42^ Interestingly, Awadalla et al.^43^ identified different miRNAs and their target genes in T1G3 UC in response to BCG. The results suggested that the overexpression of miR‐21, miR‐199a, and their target genes (STAG2 and NCOR1) predict a poor response to BCG. Rouanne et al.^44^ have investigated the mechanisms underlying BCG resistance. They found that the overexpression of human leukocyte antigen‐I hinders the immunoactive tumor microenvironment, which is associated with a favorable outcome.^44^ Kates et al.^45^ investigated the role of T cells, including effector and regulatory T cells (Tregs), in patients with BC treated using intravesical BCG. They demonstrated that BCG elevates CD4^+^ T cells, indicating that combining BCG with T cell‐activating agents might improve its clinical efficacy. In addition, they analyzed the differences between BCG responders and nonresponders to investigate the mechanisms underlying BCG resistance. Programmed death‐ligand 1 (PD‐L1) expression in baseline tumors predicts an unfavorable response to BCG.^46^ Thus, identifying eligible molecules to predict BCG response and exploring effective strategies to overcome BCG resistance is of great importance.

Currently, radical cystectomy is an alternative treatment for BCG failure, and bladder‐sparing therapies are under investigation. Combining BCG treatment with administering cytokines such as IFN‐α, IL‐2, or IFN‐γ is being evaluated. Considering that PD‐L1 is overexpressed in BCG‐unresponsive tumors and is associated with BC stage progression,^47^ several anti‐PD‐L1 and antiprogrammed cell death protein 1 (PD‐1) drugs are being tested in patients with NMIBC who are refractory to BCG, or in patients with very high‐risk NMIBC and unexposed to BCG. Due to the positive results of KEYNOTE‐057, United States Food and Drug Administration (US FDA) approved pembrolizumab for BCG‐unresponsive, high‐risk NMIBC.^48^ The NCT02808143 clinical trial assessed the safety and antitumor activity of intravesical pembrolizumab combined with BCG; the results demonstrated that the combination is safe, feasible, and elicits strong immune responses.^49^ Direct application of pembrolizumab to the bladder is a promising alternative for BCG‐unresponsive NMIBC and should be further investigated.

### 2.2 ICIs for advanced BC

Patients with NMIBC have been treated with intravesical BCG immunotherapy for more than 40 years, indicating that BC is immunoresponsive. The enthusiasm for BC immunotherapy has increased dramatically following the advent of new targeted immunotherapies, particularly ICIs.

#### 2.2.1 Effect of ICIs on immune signaling pathways on BC

**FIGURE 2:**
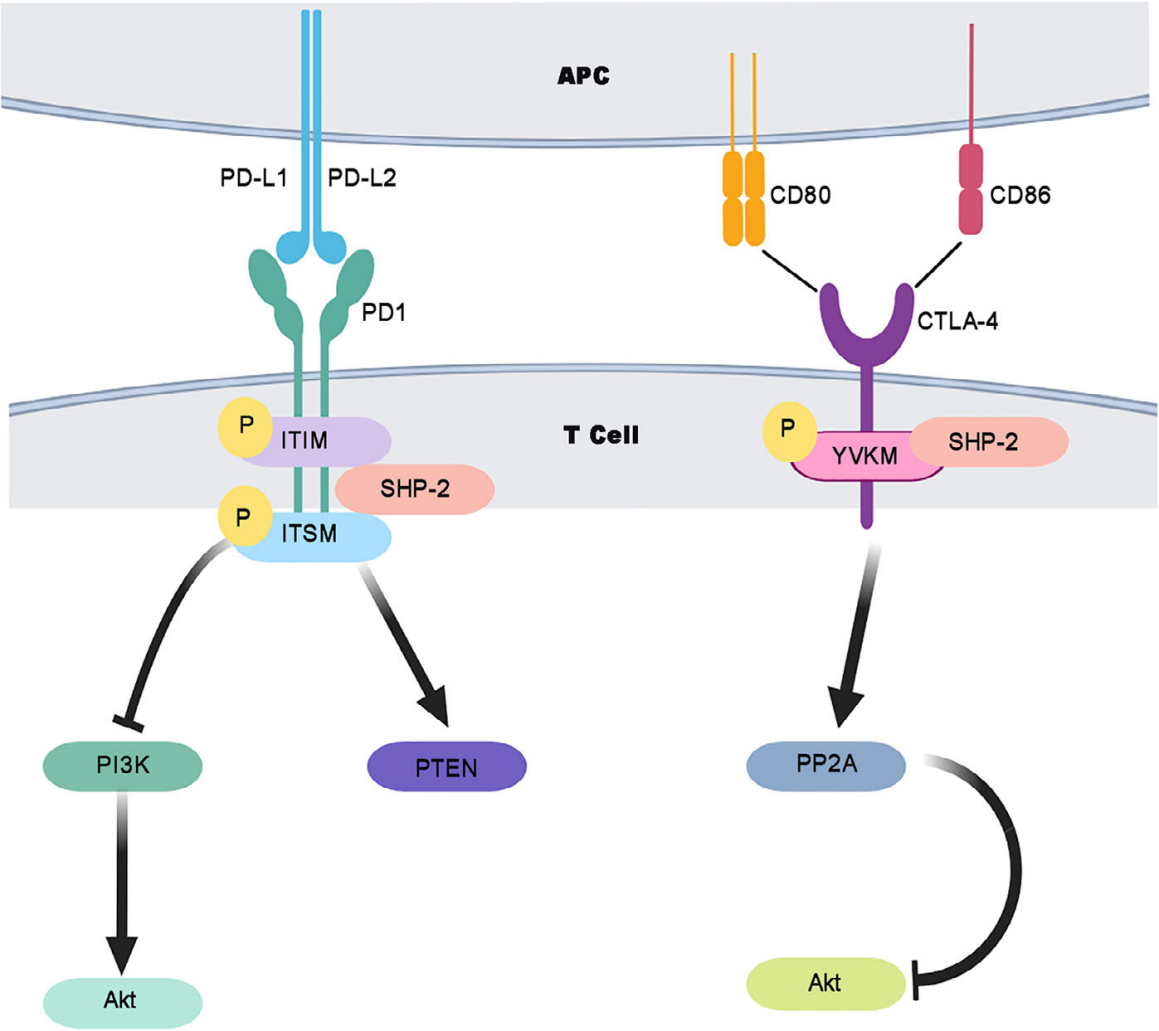
Effect of ICIs on immune signaling pathways on bladder cancer. Programmed cell death protein 1 (PD‐1) binds to its ligands PD‐L1/PD‐L2. The intracellular domain of PD‐1 becomes phosphorylated and recruits SHP2 to dephosphorylate PI3K. In addition, PD‐1 augments PTEN expression. Cytotoxic T lymphocyte‐associated protein 4 (CTLA‐4) binds to its ligands B7.1/B7.2 and can be phosphorylated by kinases. The phosphorylated CTLA‐4 inhibits AKT activity via PP2A‐dependence. APC, antigen presenting cells.

The representative immune checkpoint molecules include PD‐1, PD‐L1, PD‐L2, and CTLA‐4. In humans, PD‐1 is expressed on various activated immune cells, such as T‐cells and B cells, Tregs, activated monocytes, and myeloid DCs.^50^ It is a type I transmembrane glycoprotein that consists of a single extracellular Ig V domain, a hydrophobic transmembrane domain, and a cytoplasmic tail structure domain. PD‐L1, a ligand of PD‐1, is widely expressed in both immune and nonhematopoietic cells. In contrast, PD‐L2 is primarily distributed in activated macrophages and DCs.^51^ After PD‐L1 binding to PD‐1, two tyrosine motifs (immune receptor tyrosine‐based inhibitory motif [ITIM] and immune receptor inhibitory tyrosine‐based switch motif [ITSM]) on the cytoplasmic domain of PD‐1 become phosphorylated. Phosphorylated ITSM recruits Src homology 2 domain‐containing protein tyrosine phosphatase‐2 (SHP‐2) which dephosphorylates PI3K and other downstream signaling molecules and augments phosphatase and tensin homolog deleted on chromosome ten (PTEN) expression.^52^ Subsequently, T‐cells are exhausted. Therefore, tumor cells escape immune surveillance by upregulating PD‐L1 expression (Figure 2).

CTLA‐4 is primarily expressed by activated T cells such as CD4^+^ and CD8^+^ T cells, whereas its ligands, B7‐1 (CD80), and B7‐2 (CD86), are located on APCs including DCs, macrophages, and B lymphocytes.^53^ After ligands bind to CTLA‐4, the tyrosine YVKM motif can be phosphorylated by Src family kinases, maintaining CTLA‐4 on the cell surface to exert inhibitory function. In addition, phosphorylation of CTLA‐4 recruits SHP2 and blocks AKT phosphorylation through activating protein phosphatase 2 (PP2A).^54^ Thus, the interaction between B7‐1/B7‐2 and CTLA‐4 blocks the T cell‐mediated immune response, reduces activated T cells, and prevents the formation of memory T cells (Figure 2).

#### 2.2.2 Clinical applications of ICIs

The US FDA and European Medicines Agency (EMA) have approved five ICIs to treat metastatic BC. Up to 50% of patients with metastatic BC are ineligible for cisplatin‐based chemotherapy because of renal insufficiency, old age, or comorbidities.^55^ Pembrolizumab (PD‐1 inhibitor) and atezolizumab (PD‐L1 inhibitor) have recently been approved as first‐line therapy for these platinum‐unfit and PD‐L1 positive patients. These two drugs have been approved based on results of KEYNOTE‐052 and IMvigor210, respectively,^56^, ^57^ demonstrating comparable responses and adverse event rates.

Pembrolizumab, atezolizumab, and three other ICIs (nivolumab, avelumab, and durvalumab) have been approved as second‐line therapies for advanced or metastatic BC. Regardless of use as monotherapy or combined chemotherapy, pembrolizumab significantly improves the median overall survival (OS), with a 29% response rate.^58^ The phase 2 NCT02108652 trial showed atezolizumab significantly improved the objective response rate (ORR).^59^ No significant improvement has been observed in the OS of patients receiving atezolizumab treatment, compared with those receiving chemotherapy. However, atezolizumab treatment is safer than chemotherapy and is well‐tolerated by patients.^60^ And extending the median follow‐up of 33 months, the 24‐month OS rate was 23% with atezolizumab and 13% with chemotherapy. Safety findings were consistent with the primary analysis.^61^ These defective results made the manufacturer of atezolizumab withdraw it as a second line treatment for BC voluntarily in March 2021. In 2017, the US FDA approved nivolumab, avelumab, and durvalumab as second‐line treatments for BC. The results of CheckMate275 showed that nivolumab administration alone achieves an ORR of 19.6%, irrespective of PD‐L1 expression. Here, 18% of the patients manifested grade 3−4 adverse events, including fatigue and diarrhea. Unfortunately, three individuals succumbed to pneumonitis, acute respiratory failure, and cardiovascular failure.^62^ Findings of the clinical trial, NCT01772004, indicated the potential antitumor activity of avelumab, with a 17% ORR.^63^ Moreover, the antitumor activity of durvalumab has been clinically demonstrated against advanced urothelial BC with good safety.^64^

Ipilimumab and tremelimumab are representative drugs of anti‐CTLA‐4 antibodies. Owing to the inconsistencies in clinical trial results investigating the efficacy and safety of anti‐CTLA‐4 antibody combinations, they have not yet been approved for BC. Galsky et al.^65^ reported the clinical trial findings of NCT01524991; they demonstrated that the combination of gemcitabine, cisplatin, and ipilimumab enhances the circulation of CD4^+^ cells, which is associated with improved survival. However, the corresponding patients failed to reach the primary endpoint of 1‐year OS. Sharma et al.^66^ reported the results of CheckMate 032, an open‐label, multi‐cohort study investigating the efficacy and safety of nivolumab alone and in combination with ipilimumab in platinum‐pretreated metastatic urothelial carcinoma (mUC). Nivolumab exhibited sustained antitumor activity during long follow‐ups, either alone or in combination with ipilimumab. In addition, adverse events in these groups were manageable, suggesting that immunotherapy combinations improve urothelial cancer treatment.^66^ Einerhand et al.^67^ analyzed the differences between combined chemotherapy and immunotherapy for locally advanced urothelial cancer. The results showed that combination immunotherapy resulted in superior progression‐free survival (PFS) and OS with lower toxicity than those combination chemotherapy.^67^ Recently, de Ruiter et al.^68^ analyzed the safety of neoadjuvant immunotherapy before radical cystectomy in nonmetastatic MIBC. They found that nivolumab alone or in combination with ipilimumab is safe, and further evaluation in phase III trials is required.^68^ DANUBE, a phase III trial evaluating the OS of patients treated with durvalumab with or without tremelimumab as a first‐line therapy for mUC, did not achieve the primary endpoint of OS. No significant differences were observed between durvalumab + tremelimumab and standard chemotherapy.^69^ These negative results also caused the manufacturer of durvalumab to withdraw it as a second‐line treatment for BC voluntarily in February 2021.

#### 2.2.3 Challenges of ICI therapies

Despite the sustained responses to immunotherapy observed in clinical trials, only some patients (20−30%) respond to the therapy. The possible reasons include a lack of suitable biomarkers predicting patients, response, and immune escape.

A series of biomarkers including PD‐L1 expression, tumor mutational burden (TMB), gene expression signatures, and molecular subtypes of BC have been proposed. Although PD‐L1 overexpression is associated with advanced and aggressive tumors and poor survival outcomes, predicting the efficacy of ICIs using a single marker may lead to unsatisfactory results owing to inconsistent clinical trial findings.^20^, ^70^ TMB defines the number of mutations in a cancer sample. A published work revealed higher TMB values in responders than those in nonresponders.^71^ Using whole‐exome sequencing of RNA sequences, another work found that high TMB value is associated with a sensitive response of ICI.^72^ However, there are limitations to using TMB as a response biomarker, particularly when administered alone.^73^ Gene signatures are another well‐known biomarker for determining drug sensitivity. Recently, many novel gene biomarkers including parathyroid hormone‐like hormone, insulin‐like growth factor binding protein 7 (IGFBP7), immunogenic cell death, filaggrin, and Forkhead box Q1 (*FOXQ1*) affecting BC immunotherapy have been identified and validated using bioinformatic analyses of public databases.^74^, ^75^, ^76^, ^77^, ^78^ Based on molecular characteristics, patients with MIBC are classified into the following five subtypes: luminal papillary, luminal infiltrated, luminal, basal squamous, and neuronal.^79^ Several recent trials^80^, ^81^, ^82^ have assessed the relationship between molecular subtypes and responses to ICIs and obtained inconsistent results. Several other potential biomarkers, such as microsatellite instability,^83^, ^84^, ^85^, ^86^, ^87^ Tumor‐infiltrating lymphocytes,^88^, ^89^ T‐cell immunoglobulin and ITIM domain (TIGIT0),^90^, ^91^ and tertiary lymphoid structures^92^ are potential predictors of patient response to cancer immunotherapy.

**FIGURE 3:**
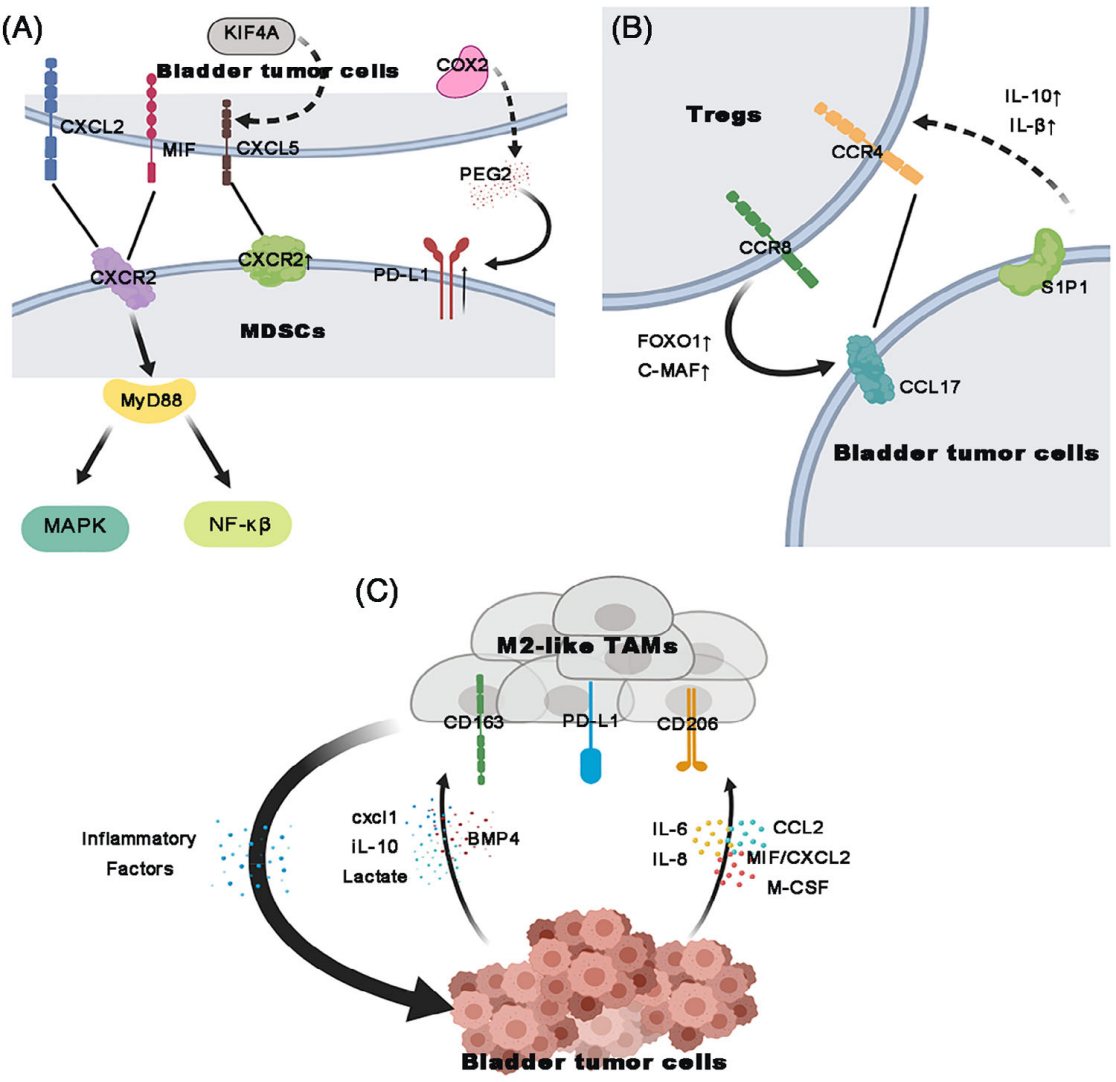
BC cells recruit various immunosuppressive cells to create an immunosuppressive microenvironment. (A) MDSCs are recruited due to different signals stimulated by BC cells. For example, KIF4A promotes the CXCL5 release; CXCL2/MIF‐CXCR2 signaling‐stimulated activation of the MAPK and NF‐κB pathways in MDSCs through MyD88; COX2/mPGES1/PGE_2_ pathway promotes PD‐L1 expression in MDSCs. (B) BC cells interact with Tregs to dampen immune response against tumors. Chemokine CCL17 interacts with CCR4 and CCR8 to induce Tregs infiltration. S1P1 activates TGF‐βand IL‐10 secretion, leading to Tregs expansion. (C) BC cells favor the M2‐like TAMs. BC cells produce chemokines to recruit TAMs. In addition, they increase CD206, CD163, and PD‐L1 levels to establish M2‐like TAMs. In return, M2‐like TAMs further support bladder tumor progression. MDSCs: Myeloid‐derived suppressor cells. Tregs, regulatory T cells; TAMs, tumor‐associated macrophages.

Another major obstacle in immunotherapy is immune escape. BC cells evade immunosurveillance by generating a complicated immunosuppressive tumor microenvironment, which alters cell components and signaling pathways. The immunosuppressive signal is amplified in the presence of immunosuppressive cells (Figure 3), such as myeloid‐derived suppressor cells (MDSCs), M2‐type tumor‐associated macrophages (TAMs) and Tregs. MDSCs accumulate in the tumor environment of human cancers. They are immature myeloid cells recruited to primary tumors and metastatic sites and inhibit innate and adaptive immune responses by suppressing CD4^+^ T cells, CD8^+^ T cells, and NK cells. Lin et al.^93^ reported that KIF4A is overexpressed in both lymph node‐positive and high‐grade BC tissues and KIF4A promotes the CXCL5 secretion to recruit MDSCs, contributing to form an immunosuppressive microenvironment. In addition, CXCL2/MIF signaling from tumor cells promotes CXCR2 expression on MDSCs, and activates the MyD88‐dependent MAPK and nuclear factor kappa B (NF‐κB) pathways, leading to MDSCs accumulation.^94^ Prima et al. demonstrated cyclooxygenase‐2/microsomal PGE2synthase 1/prostaglandin E2 (COX2/mPGES1/PGE_2_) pathway promotes PD‐L1 expression in MDSCs and pharmacologic PGE_2_ inhibitors reduce PD‐L1 levels leading to a decrease in immune suppression.^95^ Takeyama et al.^96^ investigated whether targeting MDSCs is effective against cisplatin‐resistant BC. The results showed that α‐Gr1 and α‐Ly6C antibodies significantly reduced tumor volume by increasing CD8^+^ T cell infiltration. Therefore, targeting MDSCs may enhance the efficacy of ICIs against cisplatin‐resistant BC. TAMs are present in the tumor stroma at all stages of cancer progression. They present two phenotypes called M1‐like and M2‐like TAMs. While the former inhibits tumor progression, the latter favors cancer metastasis. M2‐like TAMs prime the premetastatic site and enable tumor cell extravasation and survival, eliminate CD8 ^+^ T cells, induce Treg trafficking, and secrete immunosuppressive cytokines and bioactive lipids. BC cells produce CCL2, MIF/CXCL2, IL6, IL8, M‐CSF to recruit TAMs.^97^ In addition, they increase CD206, CD163, and PD‐L1 levels in macrophages by secreting IL‐10, CXCL1, lactate, and bone morphogenetic protein 4 to establish M2‐like TAMs.^98^ In return, M2‐like TAMs further support bladder tumor progression through increasing inflammatory factors. Thus, targeting M2‐like TAMs may enhance the efficacy of anti‐BC therapies including ICIs. With the discovery of the CD47‐signal regulatory protein‐α (SIRPα) as the “do not eat me” signaling pathway, the role of a novel subpopulation of TAMs expressing SIRPα has attracted considerable attention. Xu et al. reported that SIRPα TAMs exhaust T cells and accelerate *immune evasion*, suggesting the potential of SIRPα TAMs as therapeutic targets *in* MIBC.^99^ Tregs play an important role in the development of the immunosuppressive tumor microenvironment. They dampen chronic immune responses against tumors. Maeda et al demonstrated that Tregs is associated with poor prognosis in dogs with spontaneous BC. The interaction between chemokine CCL17 and the receptor CCR4 located on Tregs leads to Tregs infiltration. Blocking CCR4 depletes Tregs and prolongs survival.^100^ Liu et al.^101^ found that sphingosine 1‐phosphate 1(S1P1) is upregulated in BC tissues and CD4^+^Foxp3^+^ Tregs. S1P1 activates the TGF‐β signaling pathway and promotes TGF‐βand IL‐10 secretion from BC cells, resulting in Tregs expansion.^101^ Recently, CC‐chemokine receptor 8 (CCR8) has been identified as an important chemokine receptor expressed on intratumoral Tregs. In addition, Tregs are essential for CCR8 Treg‐mediated immunosuppression, wherein they upregulate the expression of *FOXO1* and *c‐MAF*.^102^ CCR8 blockade can destabilize intratumoral Tregs into a fragile phenotype, coupled with the reactivation of antitumor immunity and enhance anti‐PD‐1 therapeutic benefits in MIBC.^103^ Thus, CCR8Tregs represent a stable Treg subtype and a promising therapeutic target for MIBC immunotherapy.

**FIGURE 4:**
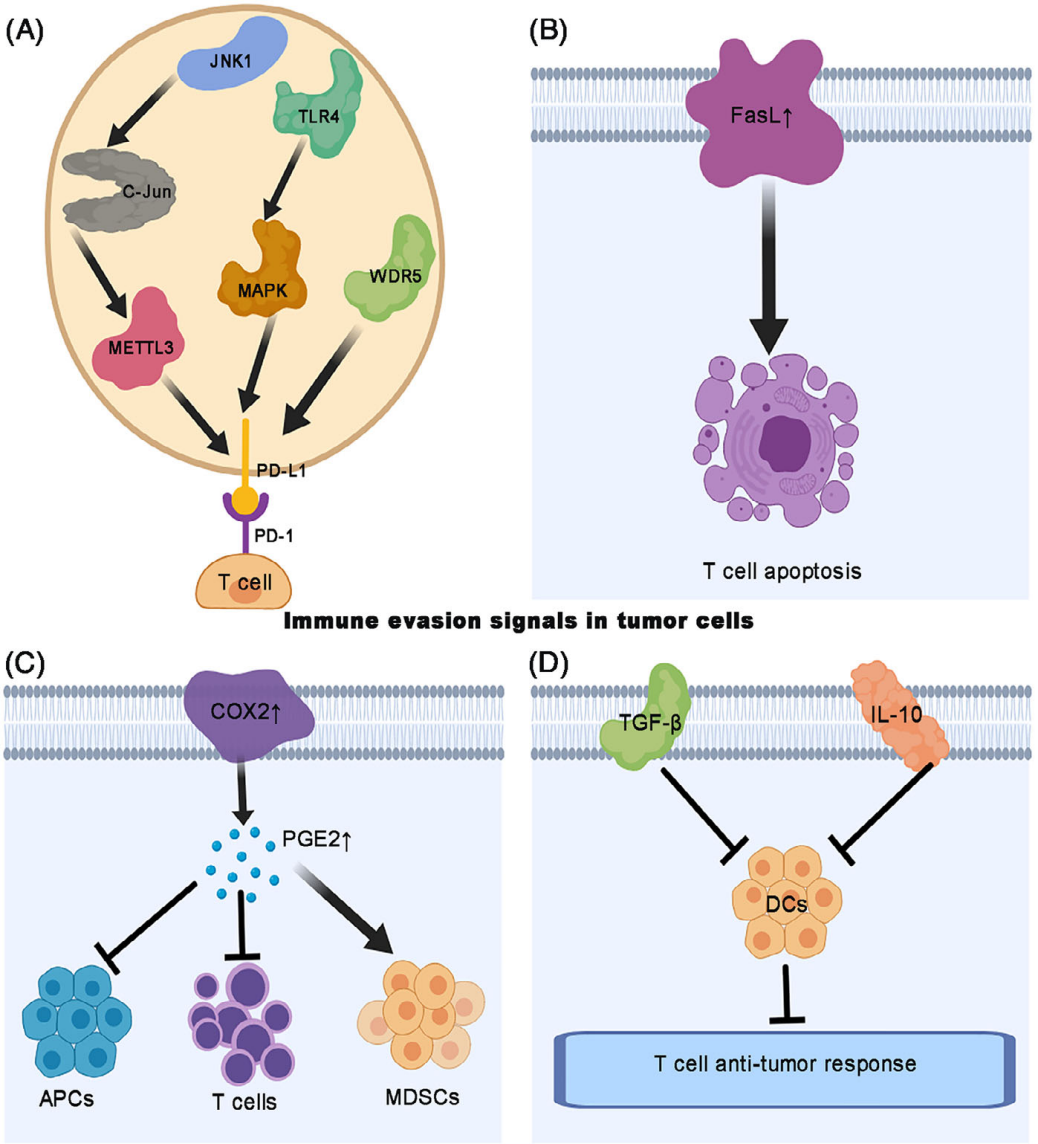
Immune escape signaling pathways in bladder cancer. (A) Multiple signaling pathways upregulate PD‐L1 expression which binds to PD‐1 on the membrane of T cells, inhibiting T cell‐mediated immune response. (B) Overexpression of FasL induces T cell apoptosis, facilitating immune escape. (C) COX2 promotes PGE2 secretion, inhibits antigen‐presenting cells and T cells, and activates MDSCs. (D) Overexpression of TGF‐β and IL‐10 inactivates dendritic cells, promoting immune escape. COX2, cyclooxygenase‐2; DCs: Dendritic cells; PGE2, prostaglandin E2.

In addition to activating immunosuppressive cells, signaling pathways play an important role in amplifying the immunosuppressive cascade (Figure 4). Tumor cells escape immunosurveillance by upregulating PD‐L1 or B7 ligands, which bind to the PD‐1 or CTLA‐4 receptors on T cells and suppress antitumor immunity. Wang et al.^104^ found that the toll‐like receptor 4 signaling pathway induces PD‐L1 expression in BC cells and promotes immune escape. However, extracellular signal‐regulated kinase (ERK) or c‐Jun N‐terminal kinase (JNK) inhibitors could block this process.^104^ RNA N6‐methyladenosine (m6A) writer methyltransferase‐like 3 (METTL3) is upregulated in BC and promotes cancer progression. Ni et al. found that JNK signaling promotes BC immune escape by regulating METTL3‐mediated m6A modification of PD‐L1 mRNA. Therefore, JNK signaling is a potential target for BC immunotherapy.^105^ WD repeat domain 5 (WDR5) positively correlates with PD‐L1 expression. In addition, OICR‐9429 (the WDR5 inhibitor) suppresses *immune evasion* by blocking IFN‐γ‐induced PD‐L1. Therefore, targeting WDR5 may enhance the antitumor effect of immunotherapy.^103^ Vidotto et al.^85^ found that *PD‐1, PD‐L1, IDO1, TIGIT, TIM‐3, TGFB1*, and *LAG3* are co‐expressed in MIBC tumors and contribute to immune evasion (Figure 4A). Furthermore, gene expression studies have demonstrated that expression of the Fas ligand (FasL) is higher in patients with BC than that in healthy individuals, regardless of the tumor grade or stage.^106^ FasL‐expressing BC cells induce the Fas‐mediated killing of autologous T lymphocytes both in vitro and in vivo. Thus, FasL facilitates immune escape by inducing T cell apoptosis (Figure 4B).^106^ The inducible inflammatory enzyme, COX2, is overexpressed in BC cells and promotes PGE2 secretion.^107^ PGE2 plays multifaceted roles in cancer progression, cancer‐related immune inflammation, and immune evasion, leading to strong antiapoptotic effects, chemotherapy resistance, and proliferation and renewal of BC stem cells.^108^ PGE2 overexpression in tumor tissues affects infiltrating immune cell functions, such as inhibiting APCs and effector T cells, and stimulating the generation of MDSCs.^109^ Moreover, Eruslanov et al.^109^ confirmed that human bladder tumors secreting PGE2 inhibit the generation of mature APCs in vitro while promoting the accumulation of monocytic MDSCs and macrophages. In summary, deregulated PGE2 metabolism in BC promotes the formation of an immunosuppressive microenvironment which supports tumor growth (Figure 4C). Many tumor‐promoting signaling molecules are involved in immune escape. Yang and Lattime found that the IL‐10‐expressing MB49 cell line prevents DCs from stimulating CD4^+^ and CD8^+^ T cell antitumor responses.^110^ Furthermore, Loskog et al.^111^ demonstrated that CD40L‐transduced MB49 cells suppress the expressions of IL‐10 and TGF‐β, promote the maturation and activation of DCs, induce a Th1‐ type response, and activate cytotoxic T lymphocytes in the tumor area. Thus, immunosuppressive signaling molecules are potential candidates for the treatment of BC (Figure 4D). ICIs restore T cell functions, and their efficacy depends on the recognition of tumor cells for destruction. Burke et al.^112^ investigated the modulation of histone deacetylases (HDAC) inhibition on cancer cell immunovisibility in heterotopic and orthotropic mouse BC. They found that HDAC inhibition eliminates the tumor “invisibility cloak” of cells which prevents T cells from recognizing and killing them. Thus, combining ICIs with locally administered HDAC inhibitors is a new approach to treat patients with BC. Xiang et al.^113^ investigated the clinical significance and immune characteristics of CCR5TINs (tumor‐infiltrating neutrophils [TINs]) in MIBC. They found that high CCR5TIN infiltration is a good predictor of OS and RFS; it may be associated with survival benefits from adjuvant chemotherapy. CCR5TINs correlate with high expressions of effector molecules in CD8^+^ T cells. Notably, pembrolizumab treatment elevates the apoptotic status of tumor cells only in the high CCR5TINs subgroup and not in the low CCR5TINs subgroup. Thus, CCR5TINs can prime the antitumor immune response by autonomous IFN‐γ release, thus leading to a favorable prognosis and superior therapeutic response to adjuvant chemotherapy and immunotherapy in MIBC.

## 3 CHARACTERISTICS OF ADCs

ADCs can inhibit the proliferation of tumor cells with specific antibody‐binding sites by delivering cytotoxins linked to monoclonal antibodies (mAbs), with high therapeutic efficacy and low toxicity, and have therapeutic potential against BC. Patients subjected to ICIs and chemotherapy are the primary targets of these drugs.^114^

### 3.1 Components of ADCs

**FIGURE 5:**
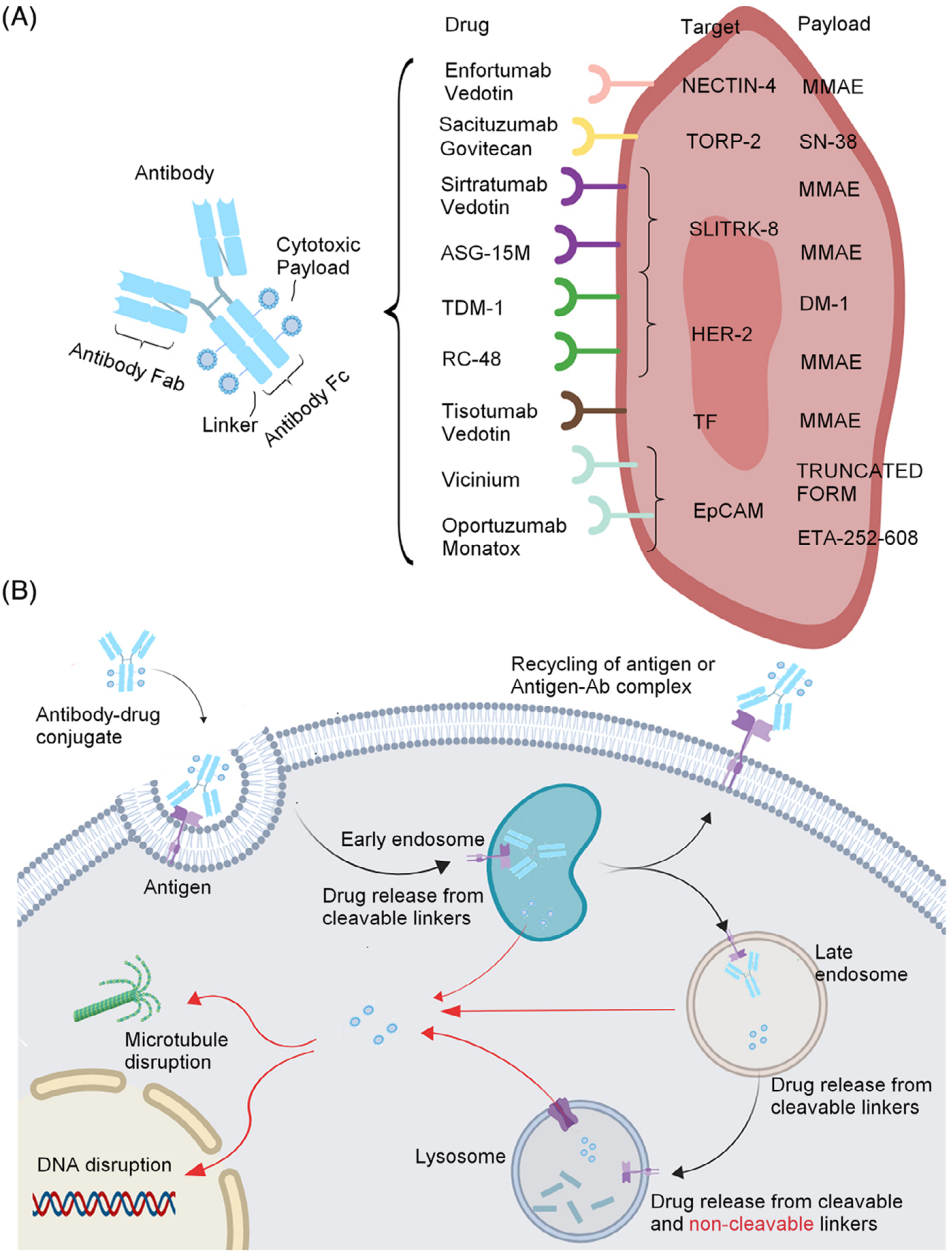
The structure of antibody–drug conjugate (ADCs) components and ADCs treatment‐associated signaling pathways on bladder cancer. (A) ADCs have three components: an antibody, a linker, and a cytotoxic payload. ADCs can identify highly expressed cellular targets using specific antibodies and hurt the tumor cells with a potent payload; the antibody or payload is different for each drug. (B) ADCs treatment‐associated signaling pathways on bladder cancer. ADCs binds to a specific antigen, endocytosed by cancer cells along with the cytotoxin. Then the ADCs payloads are detached, scatter into the cytosol, and kill cancer cells via targeting microtubules or DNA.

An ADC is a combination of an antibody, which binds to a specific antigen, a payload with potent cytotoxicity, and a connecting linker (Figure 5A). The target antigen overexpressed in cancer cells is optimal.^115^ The advantage of ADCs over previously therapies is that they can target surface proteins which are not directly related to cellular growth or proliferation to express anticancer properties.^116^ Despite its concise theoretical structure, additional factors should be considered to design an optimal ADC.

#### 3.1.1 The antibodies of ADCs

The choice of a specific target expressed in cancer cells is critical for transmitting the cytotoxic payload throughout the cells, decreasing systemic toxicities, and expanding the therapeutic window. Other factors that should be considered are limited immunogenicity and cross‐reactivity as well as strong binding affinity to the target for efficient internalization and stability.^117^, ^118^ Ideal mAbs should selectively identify antigenic targets which are strongly expressed on malignant cells.^119^ Human immunoglobulin G (IgG) is the most common antibody type, particularly IgG1, which is used in most engineered immunotherapies, including ADCs, due to its ability to stimulate immune effector function.^120^

#### 3.1.2 The linker of ADCs

The linker connects the cytotoxic payload to the antibody via a conjugation site in the antibody heavy chain. The linker must possess the following two fundamental characteristics. First, it should ensure the stability of the cytotoxic payload and antibody combination, particularly during plasma circulation, to avoid additional damage to other organs during transportation.^121^ Second, the drug should be readily released when internalized into the tumor environment.^122^ The chemical properties of the linker, including therapeutic index, pharmacokinetics, efficacy, specificity, toxicity, potency, and stability, influence ADCs capacities.^123^ Thus, an ideal linker should maintain sufficient stability in the bloodstream before reaching its target as well as in the tumor for better cancer‐specific toxicity.^124^

#### 3.1.3 The payloads of ADCs

The payload is the ultimate influencing factor in ADCs; it should have both high concentration and activity in cancer cells. Most chemotherapy is difficult to apply to ADCs due to systemic cytotoxicity and dose limitations. To overcome these limitations, the IC50 of ideal payload should be in the picomolar or nanomolar concentrations range.^125^ Therefore, highly toxic molecules are always selected as cytotoxic agents which comprise the ADCs payloads.^119^ Cytotoxic agents are classified into those damaging DNA (calicheamicin, pyrrolobenzodiazepine (PBD), duocarmycin, and topoisomerase inhibitors), and those altering tubulin formation (tubulysin, auristatin derivatives, and maytansinoids).^123^, ^125^

### 3.2 ADCs treatment‐associated signaling pathways on BC

ADCs can perform an “effective” killing effect by playing a “specific” targeting role to precisely destroy cancer cells, like a precision‐guided “biological missile,” which can reduce off‐target side effects and improve the therapeutic window.^126^ A primary ADCs treatment‐associated signaling pathway is shown in Figure 5B. Once selectively bound to a specific antigen, the antibody is internalized into cancer cells along with the bound cytotoxin, predominantly via receptor‐mediated endocytosis. After entering endosomes or lysosomes, the ADCs payloads are detached from the antibody carriers due to acidic, proteolytic, or redox conditions. They then scatter into the cytosol and attack target substrates, eventually leading to cancer cell death via targeting microtubules or DNA.^125^, ^127^ In addition, if the payload is permeable or transmembrane, its release may also enhanced the cytotoxic effects of ADCs by generating bystander effects to directly or indirectly altering the tumor microenvironment.^128^

### 3.3 Clinical and preclinical applications of ADCs in BC

ADCs can selectively deliver diverse cytotoxic drugs to cancer cells with overexpressed targets, consequently reducing toxicity, increasing efficacy, and enhancing therapeutic activity.^119^ Thus, the selection principle of ADCs is based on whether the antigens corresponding to the antibodies that make up ADCs are highly expressed in BC.^129^ Currently, ADCs is a novel therapeutic strategy for BC. For instance, the EMA and US FDA have granted EV accelerated approval for mUC treatment following the failure of ICI and platinum chemotherapy.^130^ In April 2021, the US FDA approved SG for the treatment of mUC based on preliminary data from phase I and II trials.^131^ Several other ADCs and their combinations also have been investigated (Figure 5A). Moreover, trials of the major ADCs described hereafter have shown significant efficacy, improving survival and tumor control in BC (Table 2).

**TABLE 2:** Characteristics of ADCs involved in clinical trials 2013−2022.

Clinical trial number	Drug	Target (s)	Cytotoxic agent	Development status	Objects
/	Enfortumab Vedotin	NECTIN‐4	MMAE (antimitotic drug)	US FDA approval	After failure to platinum CT and ICI
/	Sacituzumab Govitecan	Trop‐2	SN‐38 (topoisomerase IB inhibitor)	US FDA approval	After failure to platinum CT and ICl
NCT02999672	TDM‐1	HER‐2	DM‐1(topoisomerase I inhibitor)	Phase II	HER2 overexpressing locally advanced or metastatic UC
NCT05996952	RC‐48	HER‐2	MMAE (antimitotic drug)	Phase II	HER2 positive high‐risk NMIBC
NCT01963052	Sirtratumab vedotin	SLITRK‐6	MMAE (antimitotic drug)	Phase I	After failure to chemotherapy in Metastatic BC
NCT02449239	Oportuzumab monatox	EpCAM	Pseudomonas exotoxin A	Phase III	for NMIBC Previously Treated With BCG
NCT03245736	Tisotumab vedotin	TF	MMAE (antimitotic drug)	Phase II	A phase II clinical trial of TV

Abbreviations:EMA, European Medicines Agency; EpCAM, epithelial cell adhesion molecule; HER‐2, human epidermal growth factor receptor 2; SLITRK‐6, SLIT and NTRK‐like family member 6; TF, tissue factor; US FDA, The United States Food and Drug Administration.

*Data sources*—http://www.clinicaltrials.gov.

#### 3.3.1 ADCs that target Nectin‐4: EV

Nectin‐4 is a cell adhesion molecule expressed on the surface of numerous solid tumors, which is highly expressed in 79.2% of patients with UC.^129^, ^132^ Based on this property, EV, an ADC composed of a specific mAb for nectin‐4 linked to monomethyl auristatin E (MMAE), has been designed. The microtubule‐disrupting agent, MMAE, kills BC cells by causing cell death and mitotic arrest.

A global phase III randomized trial (EV‐301, NCT03474107) was conducted on EV versus chemotherapy in patients with metastatic or locally advanced disease who progressed to anti‐PD‐1/L1 therapy and platinum‐based chemotherapy. After a median follow‐up of 11.1 months, a 30% reduction in the probability of death was observed using EV compared with that with chemotherapy. The median OS significantly increased, as did PFS, disease control rate (DCR) (71.9 vs. 53.4%, *p* < 0.001), and ORR (40.6 vs. 17.9%, *p* < 0.001).^133^ Moreover, the first‐line EV‐103 trial (NCT03288545), combining EV with pembrolizumab in cisplatin‐ineligible patients with metastatic or locally advanced UC, has yielded encouraging results. Preliminary data presented at the ESMO 2019 showed a confirmed relative risk (RR) of 62%, with a 90% DCR and 14% complete response (CR) rate.^134^ As a result, EV are the first ADC approved for patients with advanced or metastatic BC who had previously been treated with platinum‐containing chemotherapy and PD‐1/PD‐L1 inhibitors. In conclusion, the approval of EV has opened a new avenue for the treatment of patients with advanced BC.

#### 3.3.2 ADCs that target Trop‐2: SG

Trop‐2 is a transmembrane glycoprotein overexpressed in various cancers. Especially in urothelial carcinoma, the proportion of Trop‐2 overexpression patients was as high as 90.3%.^129^, ^135^ SG (IMMU‐132) was designed to contain an anti‐Trop‐2 mAb linked to an active metabolite of irinotecan, SN‐38, through a hydrolyzable linker.^136^ After the phase I/II basket trial IMMU‐132‐01 (NCT01631552) demonstrated encouraging results in the phase I dose‐finding part, an open‐label, multicohort, phase II registration trial, TROPHY‐U‐01 (IMMU‐132‐06; NCT03547973), was developed to assess the influence of SG in patients with local, unresectable, or metastatic advanced UC who had progression after previous chemotherapy and platinum‐based ICI.^137^ An ORR of 27.4% (31out of 113) was observed after a median follow‐up period of 9.1 months. The CR+ partial response + stable disease for ≥6 months was 37.2% (42 out of 113) and the median duration of response (DOR) was 7.2 months [95% confidence interval (CI), 4.7−8.6 months]. The DOR ranged from 1.4 to 13.7 months in six patients who achieved CR. The median time to obtain an objective response was 1.6 months (range 1.2−5.5 months). The median OS was 10.9 months (95% CI, 9.0−13.8 months) and the median PFS was 5.4 months (95% CI, 3.5−7.2 months).^138^ Given these positive results, approval was accelerated for the application of SG in patients with metastatic or locally advanced UC who had previously received PD‐1 or PD‐L1 inhibitors and platinum‐containing chemotherapy.^139^

#### 3.3.3 ADCs that target T‐DM1and human epidermal growth factor receptor 2

In addition to the two approved ADCs mentioned above, trials are underway to evaluate numerous promising targets that are highly expressed in BC and can be designed into ADCs, such as Human Epidermal Growth Factor Receptor 2 (HER‐2) and SLIT and NTRK‐like family member 6 (SLITRK6). Ado‐trastuzumab emtansine (T‐DM1) is an ADC which selectively delivers the microtubule poison emtansine (DM1) to target cells using the mAb, trastuzumab. The US FDA approved T–DM1 for the treatment of patients with advanced breast cancer based on the encouraging results of the tZXhe EMILIA phase III randomized trial, which showed that T‐DM1 was superior to lapatinib/capecitabine in terms of PFS and OS.^140^ In BC, Hayashi et al.^141^ compared the effects of T‐DM1 versus trastuzumab in two patients who overexpressing HER2. T‐DM1 resulted in greater growth inhibition than that with trastuzumab in the highest HER2‐expressing BC cell line, RT4V6, and improved antitumor effects in preclinical models of HER2‐overexpressing BC.^141^ Another HER2‐targeting ADC under investigation, disitamab vedotin (RC48‐ADC), could deliver MMAE to tumors through a novel humanized anti‐HER2 antibody with a cleavable linker. A phase I trial of four mUC patients with HER2‐overexpressed (IHC 2+ or 3+) was organized. This ADC demonstrated excellent antitumor activity in mUC, with a DCR of 100% and RR of 50%.^142^ Therefore, ADCs targeting HER2 may bring hope for BC in the future.^143^, ^144^

#### 3.3.4 ADCs that target SLITRK6: Sirtratumab vedotin

Results from more than 500 tissue microarrays have revealed that approximately 88% of BC specimens express SLITRK6.^145^ Hence, sirtratumab vedotin was developed to deliver MMAE to cells expressing SLITRK6 by connecting specific human antibodies via a protease cleavable linker.^145^ In a phase I dose escalation study (NCT01963052), 42 evaluable patients were treated with sirtratumab vedotin at doses of 0.5 mg/kg, which was considered active; the ORR was 33%, including 5 of 12 patients (42%) with previous treatment failure of CPIs and 4 of 11 patients (36%) with liver metastases.^146^

#### 3.3.5 ADCs that target EpCAM: Oportuzumab monatox

In addition to ADCs, peptide–drug conjugates (PDCs) are a novel class of prodrugs that can selectively deliver a payload through a sequence‐specific peptide inside or bonded to the tumor surface. Epithelial cell adhesion molecule (EpCAM) is overexpressed in several epithelial tumors, particularly UC.^147^ One PDC, oportuzumab monatox (OM), is a recombinant fusion protein composed of a single‐chain antibody, humanized anti‐EpCAM, and a truncated version of *Pseudomonas* exotoxin A,^148^ which blocks protein synthesis and causes cancer cell death.^147^ Currently, Sesbio has filed an US FDA application for the use of OM in the treatment of NMIBC which is unresponsive to BCG.^119^

#### 3.3.6 ADCs that target tissue factor: Tisotumab vedotin

Tissue factor (TF) is a transmembrane glycoprotein that plays a vital role in cancer angiogenesis, growth, metastasis and acts as the initiator of TF in the coagulation pathway.^149^ Targeting TF with immunotherapy is promising in the field of cancer treatment, and its potential efficacy and safety have been proven. Moreover, TF is effective against BC. Thus, the use of TF as an antibody to design ADCs is a feasible BC treatment strategy. The first‐in‐human ADC, tisotumab vedotin (TV), is composed of a human mAb directed against TF and MMAE via a protease‐cleavable valine‐citrulline linker. De Bono et al. conducted a phase I/II clinical trial of TV in patients with BC, wherein four patients (26.7%) achieved a confirmatory objective response on the Response Evaluation Criteria of Solid Tumors (version 1.1).^150^ In various BC solid tumors, TV has demonstrated promising preliminary anticancer activity.^150^

### 3.4 Challenges and future opportunities of ADCs

Following mAbs, ADCs have emerged as prospective highly potent drugs, which are conducive to improving the treatment outcomes and prolonging the lifespan of cancer patients. Nevertheless, ADCs possess their narrow limitations and challenges in structural design and clinical development likewise. Additionally, the appearance of ADCs resistance is a tough nut to crack in cancer therapy.^151^, ^152^ Many cell surface antigens are covered by barriers such as mucins or hyalurons or removed by cancer cells^153^ so that mAb of ADCs cannot bind with it, which gives rise to resistance in tumor cells. Some other reasons for ADCs resistance include downregulation of the antigen levels, upregulation of drug‐efflux pump, mutations of the receptors combined with targets, inhabitation of apoptosis via upregulating of antiapoptotic molecules like BCL‐2; transformation of signaling pathways etc.^153^ To surmount this, dual drug delivery of ADCs can be designed via a hetero‐functional linker which provides site specificity and multidrug loading. Such linker involves N‐aryl maleimide for the sake of providing flexibility to concatenate two different drugs.^154^ Combining two distinct antibodies through dual payload ADCs, is probable to have an antagonistic effect on drug resistance.^155^ Moreover, several preclinical and clinical reports have shown that ADCs in combination with immunotherapy can also combat resistance through two approaches, additional mutual interaction with immune cells or induction of tumor‐specific adaptive immunity. The union of them can increase the overall prognosis of patients. However, the detailed research on optimal dose, and mechanism of synergistic action are yet to be explored.^156^

It is precisely many pharmacokinetic factors that lead to a narrow therapeutic index for ADCs, which is another big challenge during the clinical development. Numerous ADCs generate toxicity on account of the uncoupling of linkers or antibodies, just as observed in PBD deconjugated with disulfide linker resulting in neurotoxicity.^157^ Based on the available reports, clinical trials of marketed ADCs such as Trodelvy, Padcesv, and so on have documented the occurrence of mild to moderately severe neutropenia, alopecia, and gastrointestinal adverse effects.^158^ The current focus of research on designing future ADCs revolves around enhancing individual ADCs components, seeking more stable linker technology for systemic circulation, investigating alternative conjugation or targeting approaches, increasing drug loading capacity, exploring novel ADC targets, etc. to reduce toxicity. Modifying the linker is the best approach, as it is critical to the release of the payload to cancer. Besides, the stability and toxicity of ADCs are related to it. For the optimization of ADCs, it is vital to the physicochemical property of the linker. The hydrophilicity of the linker can be commonly controlled through two methods; PEG or sulfonate moiety incorporated linker and having a charged group within the linker. In a recent study conducted by Zacharia et al.^159^ in 2022, they explored to development of homogeneous THIOMAB ADCs through the merging of the XTEN polypeptide scaffold with cysteine‐engineered THIOMAB antibodies. The hydrophilic XTEN polypeptide served as an alternative to PEG, which resulted in ADCs exhibiting DAR of up to 18 and a lengthened half‐life.^159^ Hopefully, with the development of ADCs, more benefits can be provided for the treatment of BC.

## 4 TKIs IN BC

**FIGURE 6:**
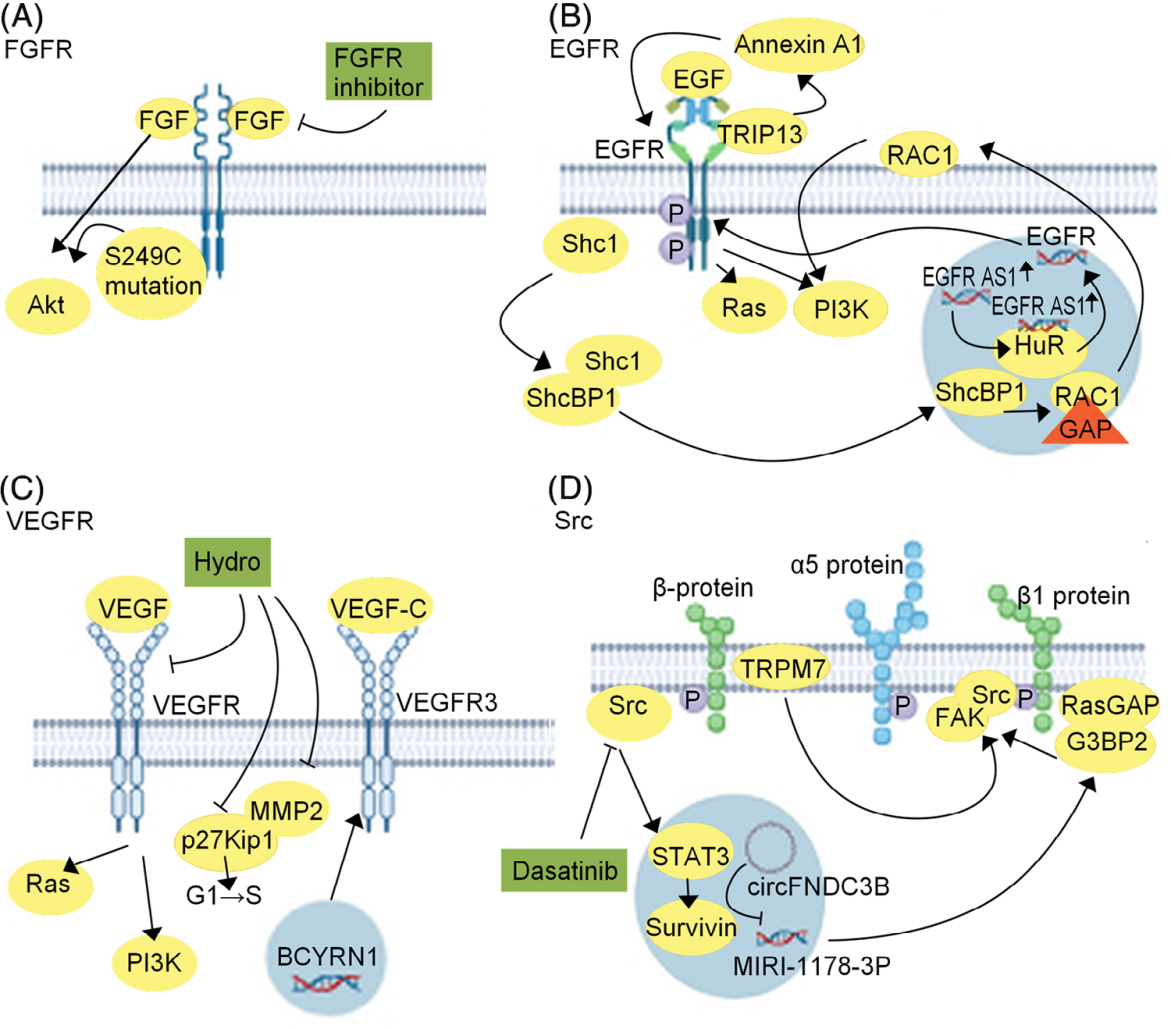
The tyrosine kinase inhibitors (TKI) therapy in bladder cancer. (A) FGFR, (B) EGFR, (C) VEGFR, and (D) Src are the representative tyrosine kinases studied in BC. The protein molecules on these signaling pathways are potential targets for bladder cancer treatment and new targeted drugs corresponding to these targets are being investigated. GAP, GTPase‐activating protein.

Targeted drugs such as TKIs are critical molecules in cancer treatment. The first oral small‐molecule inhibitor, erdafitinib, which targets FGFR3, was approved in 2019 for the Third‐line treatment of BC bearing susceptible FGFR2/3 mutations or for the second‐line treatment of locally advanced or metastatic BC following platinum‐based chemotherapy.^160^ Clinical trials evaluating the efficacy and safety of FGFR, VEGFR inhibitors, and other TKIs for BC are currently in progress (Table 3) and these drugs are expected to be approved in the near future. Here, we summarize the application status of TKIs in BC and combine it with related basic research, including the relevant signaling pathways (Figure 6).

**TABLE 3:** Characteristics of TKIs included in clinical trials among 2008−2022.

Clinical trial number	Drug	Target(s)	Development status	Objects
NCT02365597	Erdafitinib	FGFR	US FDA approval	Metastatic or unresectable UC
NCT02393248	Pemigatinib	FGFR	Phase II	Various solid tumors, including UC
NCT01004224	BGJ398	FGFR	Phase I	FGFR3‐modified metastatic UC
NCT01976741	Rogaratinib	FGFR	Phase I	Advanced UC who had previously received platinum chemotherapy and had FGFR changes
NCT02278978	Nintedanib	FGFR, VEGFR, PDGFR	Phase II	Patients with advanced FGFR3 overexpressed who have failed platinum‐based chemotherapy
NCT03827837	Famitinib	VEGFR‐2, PDGFRβ, c‐kit	Phase II	Advanced or metastatic UC after platinum‐based chemotherapy
NCT01844947	Sorafenib	RAF/MEK/ERK, VEGFR, and PDGFR	Phase I	Platinum‐based progressive metastatic UC
NCT01118351	Sunitinib	VEGFR	Phase II	NMIBC after Bacillus Calmette‐Guérin treatment
NCT03023319	Bosutinib	Src/Abl, c‐KIT, PDGFR	Phase I	Solid tumors, including those with UC

Abbreviations: ERK, extracellular regulated protein kinases; FGFR, fibroblast growth factor receptor; PDGFRβ, platelet‐derived growth factor receptor β; Src, steroid receptor coactivator; VEGF, vascular epithelial growth factor.

*Data sources*—http://www.clinicaltrials.gov.

### 4.1 Clinical studies on inhibitors targeting FGFR pathways in BC

Erdafitinib, an FGFR3 inhibitor, has been approved for the treatment of metastatic or unresectable UC based on the results of the global open‐label phase II study BLC2001(NCT02365597).^161^ The trial findings revealed that PO_4_ concentration has a guiding effect on the therapeutic dose of Erdafitinib.^162^ In addition, the ORR of patients was 40%, and no safety concerns were reported during long‐term follow‐up. Erdafitinib has demonstrated satisfactory efficacy and safety in patients with locally advanced or metastatic UC with FGFR changes (Figure 6A).^163^ Also, pemigatinib is a highly effective FGFR 1−3 selective oral inhibitor. The Phase I/II FIGHT‐101 study (NCT02393248) found that pemigatinib exerts upstanding therapeutic effects in patients with various solid tumors, including those in UC, with a median DOR of 7.3 months.^164^ Moreover, BGJ398 is an oral FGFR1‐3 inhibitor with bioavailability, selectivity, and adenosine triphosphate (ATP) competition. A prospective evaluation study showed a good therapeutic effect of BGJ398 in patients with FGFR3‐modified mUC, with a DCR of 64.2% and a total remission rate of 25.4%.^165^

Another FGFR inhibitor, rogaratinib, a pan‐FGFR inhibitor, has demonstrated promising therapeutic activity against advanced UC in phase I clinical trials.^166^ A randomized, open‐label phase II/III clinical trial has shown good therapeutic efficacy and safety of rogaratinib in patients with advanced UC who had previously received platinum chemotherapy and had altered FGFR levels. The total effective rate was 20.7%, the median OS time was 8.3 months, and the incidence of grade 3/4 adverse events was 43.0 and 4.7%, respectively.^167^

### 4.2 Clinical studies on inhibitors targeting EGFR in BC

EGFR has been dubbed the “golden target” in the era of targeted therapies. At the nucleic acid level, the role of EGFR in the occurrence and development of cancer is closely related to some long‐chain noncoding RNAs (lncRNAs). EGFR‐AS1 is a lncRNA linked to renal cancer metastasis. It maintains EGFR mRNA stability and promotes the proliferation and metastasis of renal cancer cells by binding to the RNA‐binding protein HuR.^168^ Recently, EGFR‐AS1 overexpression has been observed in BC tissues and is predictive of poor prognosis in patients.

As important membrane surface receptors, other membrane proteins are likely to play important roles in promoting EGFR‐related cancer. Furthermore, EGFR‐AS1 promotes BC metastasis by inhibiting EGFR mRNA degradation and increasing EGFR expression. Specifically, the EGFR‐AS1/EGFR pathway may be a diagnostic marker and therapeutic target in BC.^169^ Li et al.^170^ reported a close association between high annexin A1 expression level and the progression and poor prognosis of patients with BC. Annexin A1 promotes the proliferation and migration of BC cells by activating EGFR signals and its downstream signaling pathways, suggesting its role as a promising biomarker for BC prognosis. In addition, EGFR interacts with hormones in the human body to promote the occurrence and development of cancer. Elevated thyroid hormone receptor interactor 13 (TRIP13) levels have been reported in patients with BC. Mechanistic studies have demonstrated that TRIP13 directly binds to EGFR and regulates the EGFR signaling pathway, thereby influencing the stage, lymph node metastasis, distant metastasis, and survival rate of patients with advanced BC. Thus, TRIP13 is a novel biomarker and target for BC treatment.^171^

There are abundant signaling pathways downstream of EGFR, and identifying new and important signaling pathways may provide powerful breakthroughs in targeting EGFR to treat BC. EGF/EGFR activation results in the separation of SHC‐binding protein 1 (SHCBP1) from SHC adaptor protein 1 (SHC1), which then translocates to the nucleus and promotes cancer development via multiple signaling pathways.^172^ Yin et al.^173^ identified the EGF–SHCBP1–RACGAP1–RAC1 axis, revealing that SHCBP1 is upregulated in the tissues and cells of BC. Following Ser273 phosphorylation, the released SHCBP1 responds to EGF stimulation by transferring it to the nucleus. Additionally, SHCBP1 also promotes cell migration by inhibiting RACGAP‐mediated GTP‐RAC1 inactivation (Figure 6B).^173^ Although EGFR is a classic target, it can be extended to large research projects from the perspective of signal networks. Nucleic acids, proteins, hormones, and new signaling pathways together constitute the regulatory network of the system, with EGFR as an important node. Thus, we can gradually and clearly understand EGFR targets and promote the generation of anticancer drugs and strategies targeting EGFR.

### 4.3 Clinical studies on inhibitors targeting VEGFR in BC

Combining TKIs targeting VEGFR with neoadjuvant chemotherapy could be a viable treatment option for local MIBC.^174^ A phase II clinical trial used neoadjuvant gemcitabine and cisplatin chemotherapy combined with the VEGFR TKI inhibitor Nintedanib or placebo to treat locally advanced MIBC. The results show that it is safe to add Nintedanib to chemotherapy for MIBC, but it does not improve the complete pathological remission rate.^175^ Famitinib is targeted against stem cell factor receptor (c‐kit), VEGFR‐2, and PDGFRβ, and it has antiangiogenesis and antitumor cell proliferation activities, while Camrelizumab is a mAb against PD‐1. An open, multi‐center phase II clinical trial of Camrelizumab and famitinib (NCT03827837) included 18 patients with advanced or metastatic UC after platinum‐based chemotherapy. Patients who received combination therapy achieved an objective efficacy rate of 38.9% and a median PFS time of 8.3 months. Thus, famitinib combined with Camrelizumab exhibits strong antitumor activity.^176^ However, highly efficient treatment options for platinum‐based progressive mUC are still lacking.^177^ A Phase I clinical trial investigated the feasibility of combining the microtubule inhibitor, vinflunine, with sorafenib as second‐line therapy in 22 patients with platinum‐based progressive mUC. The results showed an overall response rate of 41% in this combination regimen. The trial results preliminarily confirmed the dose range, efficacy, and safety of the drug combination.^178^ Sunitinib is a new multitarget oral tumor treatment drug which can significantly inhibit VEGFR and is considerably effective in BC.^179^ A single‐arm phase II clinical study demonstrated the safety of sunitinib in patients with NMIBC who failed to receive BCG treatment, but the study did not provide information regarding improvement in the clinical outcome.^180^ Hydra, is a closed coumarin can potentially treat BC by inhibiting VEGF‐stimulated angiogenesis. The drug targets p27kip1‐dependent G1 cell cycle arrest, VEGFR‐2‐mediated signal transduction, and MMP‐2 expression.^181^ BCYRN1 is suggested as a new therapeutic target for BC (Figure 6C).^182^

Research on VEGFR‐targeted therapy for BC focuses on the existing new clinical application strategies of VEGFR inhibitors, and a combination of drugs is a valuable choice for patients with BC who have previously received chemotherapy. However, based on the results of existing clinical trials, using drug combinations has obvious safety advantages. However, it cannot achieve the ideal curative effect, and the development of new drugs may break through the existing predicament.

### 4.4 Clinical studies on inhibitors targeting Src in BC

Proto‐oncogene tyrosine‐protein kinase SRC (SRC) is a nonreceptor tyrosine kinase which can be activated by many signal transduction pathways. Bosutinib and dasatinib can significantly inhibit the conduction of SRC proteins and related signaling pathways and have been clinically proven to have good activity in various solid tumors.^183^ A Phase I dose escalation study involving 12 patients with solid tumors, including patients with UC, investigated the safe dose and adverse effects of Bosutinib combined with the antifolate drug, pemetrexed. The results showed a maximum tolerated bosutinib dose of 300 mg/day and a disease stability rate of 58% after treatment.^184^

Focal adhesion kinase (FAK) regulates tumor invasion and metastasis by binding to the steroid receptor coactivator. The knockout of G3BP2, a member of the Sh3 domain‐binding protein (G3BP) family of RasGAP enzyme activator proteins (Rasgap), limits the migration and invasion of human lung cancer cells by inhibiting the activities of SRC and FAK.^185^ Liu et al.^186^ found that invasion‐associated cyclic RNA circFNDC3B inhibited BC progression via the MIRI‐1178‐3P/G3BP2/SRC/FAK axis. TRPM7 is a member of the transient receptor potential (TRP) channel family, which regulates the migration and invasion of BC cells via the Src, Akt, and JNK signaling pathways. TRPM7 inhibitors reduce tumor size in xenotransplantation models (Figure 6D). Therefore, TRPM7 inhibition may be a promising treatment option for patients with BC.^187^

### 4.5 Challenges of TKIs and potential solutions

As for TKIs that have not currently been approved for BC, the challenges include the following aspects: (i) The molecular mechanisms of the identified therapeutic targets are not fully understood: for some potential TKI drugs, the molecular mechanism research is mainly biased towards classical signaling pathways and did not explore new pathways.^169^, ^188^ (ii) The identification of new therapeutic targets has lagged: most of the studies did not explore the upstream targets, mainly downstream of the classic TKI targets.^173^, ^187^ (iii) Trials on the anti‐BC activities of small‐molecule natural drugs have stagnated in the preclinical stages. Most of the activity studies of small molecule natural drugs are limited to effects on conventional signaling pathways.^189^, ^190^

Thus, elucidating molecular characteristics of signal proteins, screening potential natural compounds, combining TKIs with clinical chemotherapeutic drugs, and accelerating existing clinical trials are meaningful to improve BC treatment.

## 5 METABOLIC DYSFUNCTION IN BC

**FIGURE 7:**
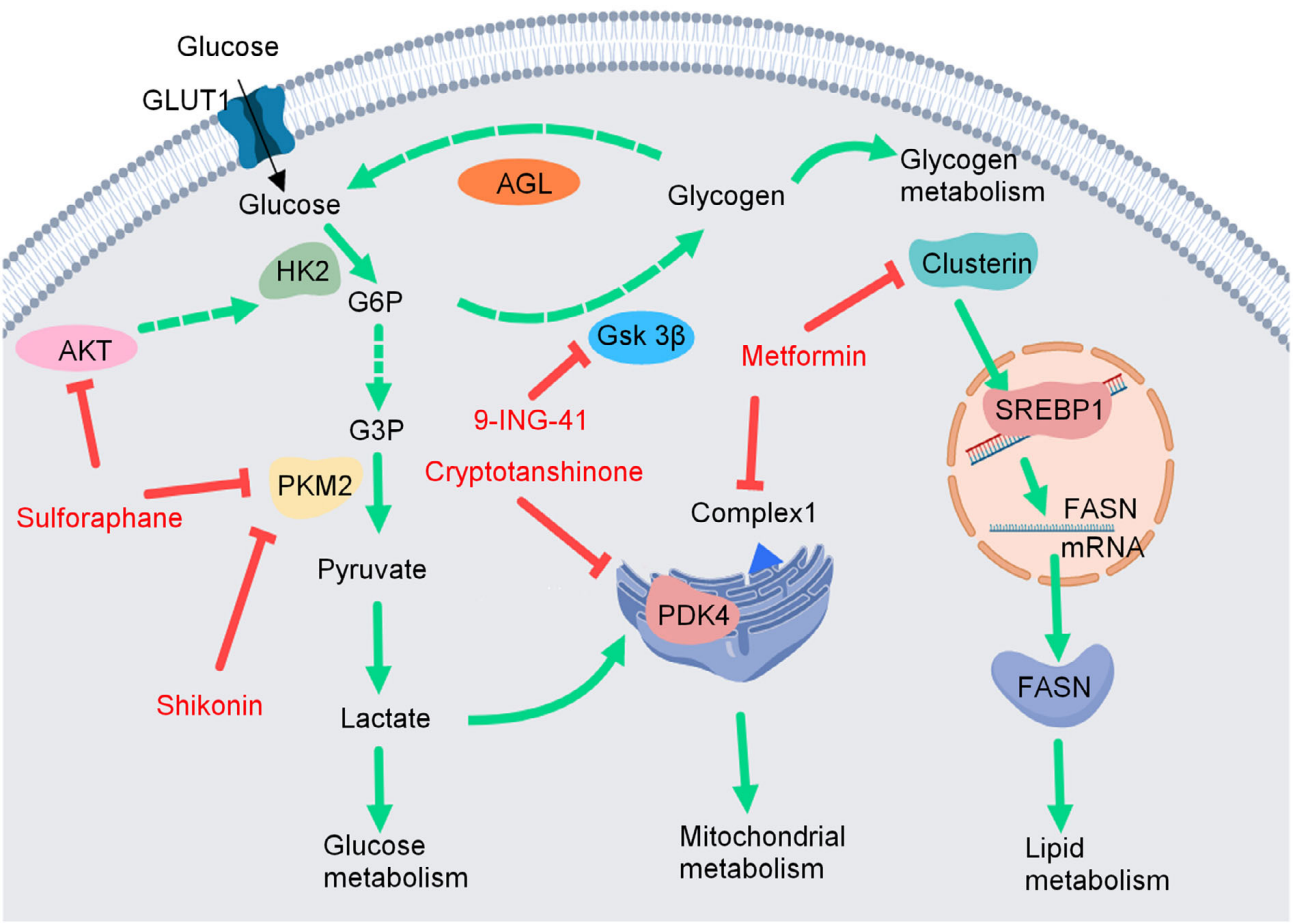
Metabolism pathways and drug targets in bladder cancer. Metabolism dysfunction is a hallmark of cancer. Glucose, mitochondrial, and lipid metabolisms are explored in bladder cancer. Multiple small‐molecule inhibitors against different receptors and signal proteins associated with these metabolism pathways have been tested in preclinical and clinical studies.

Tumorigenesis and progression are characterized by unique metabolic reprogramming which maintains the high proliferation of cancer cells.^16^ Metabolic dysfunction is a hallmark of cancer and has gained considerable attention. In BC, alterations in multiple metabolic signaling pathways, including glucose, mitochondrial, lipid, and glycogen metabolic pathways, have been observed. Metabolism is mediated and controlled by a series of enzymes; these have attracted considerable attention and are expected to be potential targets for BC therapy. Here, we summarize several metabolic pathways that play important roles in BC, with a focus on signaling pathway changes and candidate drugs targeting metabolism (Figure 7).

### 5.1 Signaling pathways and drug development of glucose metabolic dysfunctions in BC

Glucose metabolic disorders are key features of cancer cells. The expression levels of pivotal enzymes and transporters involved in aerobic glycolysis have been evaluated as prospective therapeutic targets.^1^, ^191^ Many oncogenic drivers including *GLUT1, c‐Myc, PI3K, KRAS, Akt, PTEN*, and *HIF‐1α* activate and upregulate glycolytic genes involved in BC.^192^ GLUT1 increases glucose uptake and glycolysis rate, and excess glucose is eventually converted to excess pyruvate. Pyruvic acid is transported to the mitochondria by mitochondrial pyruvate carriers. In the mitochondrial matrix, pyruvic acid is converted into carbon dioxide and water through the tricarboxylic acid cycle and coupled electron transfer chain, producing a large number of ATP molecules. Additionally, lactate dehydrogenase (LDH) catalyzes the conversion of pyruvate to lactic acid in the cytoplasm.^193^ While the pyruvate dehydrogenase (PDH) complex metabolizes pyruvate in the mitochondria, PDH kinase 1−4 largely blocks this interaction. Sulforaphane downregulates aberrant independent aerobic glycolysis in BC by blocking the AKT1/HK2 axis and PDH expression.^194^ Treatment with a combination of α‑lipoic acid and hydroxycitrate inhibits cancer cell proliferation and improves survival in mice with bladder tumor xenografts by inhibiting glycolysis and lipid synthesis, respectively.^195^ In addition to increased glucose uptake, the levels of some regulatory genes involved in glucose metabolism, such as PFKFB4, hexokinase 2, and pyruvate kinase 2 (PKM2), are upregulated in BC tumors, compared with those in normal epithelial cells.^196^ Shikonin is a PKM2 inhibitor that overcomes cisplatin resistance in BC by inducing necroptosis.^197^ Furthermore, Su et al.^198^ found that inhibiting PKM2 enhances the anticancer efficiency of pirarubicin in BC. Similarly, inhibiting the rate‐limiting enzyme, HK2, during glycolysis suppresses the proliferation of BC cells by reducing glycolysis.^199^ The use of glycolysis‐related proteins as therapeutic targets is an effective strategy for treating BC, and several glycolysis‐related protein inhibitors have been effective in eliminating BC.

### 5.2 Signaling pathways and drug development of mitochondria‐metabolic dysfunctions in BC

Mitochondria are central metabolic organelles that serve as sites for biosynthesis and are responsible for ATP production. Alterations in mitochondrial metabolism are common in cancer and can considerably affect the synthesis and bioenergetics of metabolites.^200^ Mitochondrial dysfunction exists in BC.^200^, ^201^ Several mitochondrial proteins, such as isocitrate dehydrogenase,^202^ Lon protease,^203^ and PDK4^196^, ^204^ are abnormally expressed in BC. Targeting mitochondrial metabolism‐related proteins is a potential therapeutic strategy. Lon protease inhibition reduces mitochondrial function and glycolysis, and inhibits cell proliferation, indicating that mitochondrial oxidative phosphorylation is crucial for cell growth.^205^ Pereira et al. reported that a third‐generation photosensitizer, ChlGal8, which is a chlorin conjugated with galactodendritic units, exhibits therapeutic effects in BC.^206^ Cryptotanshinone, a PDK4 inhibitor, reduces the invasion and metastasis of BC cells via the mTOR/β‑catenin/N‑cadherin pathway.^207^ Silencing PDK4 in BC cell lines retards cell migration and invasion. PDK4 plays a key role in the growth and metastasis of BC cells by altering the expression of ERK, SRC, and JNK.^208^ Research indicates that metformin prolongs the survival of patients with BC.^209^, ^210^ Metformin and other biguanides are activators of adenosine monophosphate‐activated protein kinases, which stimulate many metabolism‐related signal pathways^211^; it also inhibits mitochondrial complex 1. Preclinical studies have demonstrated that metformin inhibits the growth of BC cells mainly through the Akt‐, mTOR‐, and ERK‐related signaling pathways and potentially by limiting stemness. Although laboratory research indicates that metformin plays a potential role in the treatment of NMIBC, sufficient clinical trials to verify this finding are lacking. Nonetheless, an early multicenter, open‐label, phase II clinical trial is in progress. All 49 patients with low‐grade NMIBC were monitored after 3 months of metformin treatment. The primary outcome is the overall response of the patients, whereas the secondary outcomes are the time to recurrence, proximity to metformin, and partial response. The clinical trial is scheduled to be completed by 2025; however, no results have yet been released. The positive results may provide substantial evidence for the use of metformin in the management of NMIBC.^212^ Although preclinical research has a solid foundation, further clinical trials are warranted to explore the potential role of metformin in BC treatment. Metformin and other biguanide derivatives have enormous anticancer potential and require further investigation.

### 5.3 Signaling pathways and drug development of glycogen metabolic dysfunctions in BC

Altered glycogen metabolism is often associated with cancer development.^213^, ^214^ Amylo‐alpha‐1‐6‐glucosidase‐4‐alpha‐glucanotransferase (AGL) may act as a tumor suppressor in BC. Patients with low AGL tumor mRNA expression have poor survival outcomes, suggesting that low AGL expression promotes tumor growth after xenotransplantation.^213^ In addition, GSK‐3 β is a serine/threonine protein kinase known to regulate protein structure and transcription factors, as well as cell differentiation, proliferation, survival, and apoptosis. It is considered a therapeutic target for many major diseases, such as cancer, neurodegenerative diseases, and neuropsychiatric diseases. The efficacy of 9‐ING‐41, a GSK‐3 β inhibitor, has been evaluated in patients with advanced BC through a phase I/II clinical trial.^215^ Treatment with 9‐ING‐41 enhances the antitumor effects of chemotherapeutic drugs and improves their cytotoxic effects on human immune cells in BC cell lines.

### 5.4 Signaling pathways and drug development of lipid metabolic dysfunctions in BC

Lipid metabolism influences the progression and treatment of cancer. However, few studies have explored the relationship between lipid metabolism and BC.^216^, ^217^ A comprehensive bioinformatics study has shown that genes related to lipid metabolism affect the prognostic outcome and function of high‐level BC. A series of new prognostic markers have been identified, which contain 11 genes, including *FASN, MBOAT7, SERPINA6, PPAR GC1B, FADS1, CPT1B, HSD17B1, OSBPL10, AKR1B1, CCDC58*, and *PLA2G2F*, involved in lipid metabolism.^217^ Deng et al.^218^ reported that clusterin promotes the growth of BC and metformin targets clusterin to inactivate SREBP‐1c and its downstream target, FASN, resulting in the inhibition of de novo fatty acid synthesis, consequently inhibiting tumor growth in BC. In addition, *MEX3C* is a novel oncogene involved in the regulation of lipid metabolism through the MAPK/JNK pathway, promoting the occurrence of BC.^219^ Thus, lipid metabolism may be a promising target for the treatment of BC.

## 6 THE CHARACTERISTICS OF BC STEM CELLS

CSCs are slow‐growing and self‐renewing subsets of solid tumors that are related to tumor occurrence, development, drug resistance, and recurrence. They have existed in the tumor microenvironment for a long time^17^ and are present in many tumor types, including BC. In 2009, researchers first described CSCs in BC. Several studies have investigated the targets of BC stem cells (BCSCs). These markers, including CD44, CD24, and CD133, have been used for targeted therapy.^18^ In addition, molecules such as SOX, Oct4, Nanog, and ALDH, can also be used as BCSC markers and are expected to be used in future targeted therapies.^220^, ^221^, ^222^

**FIGURE 8:**
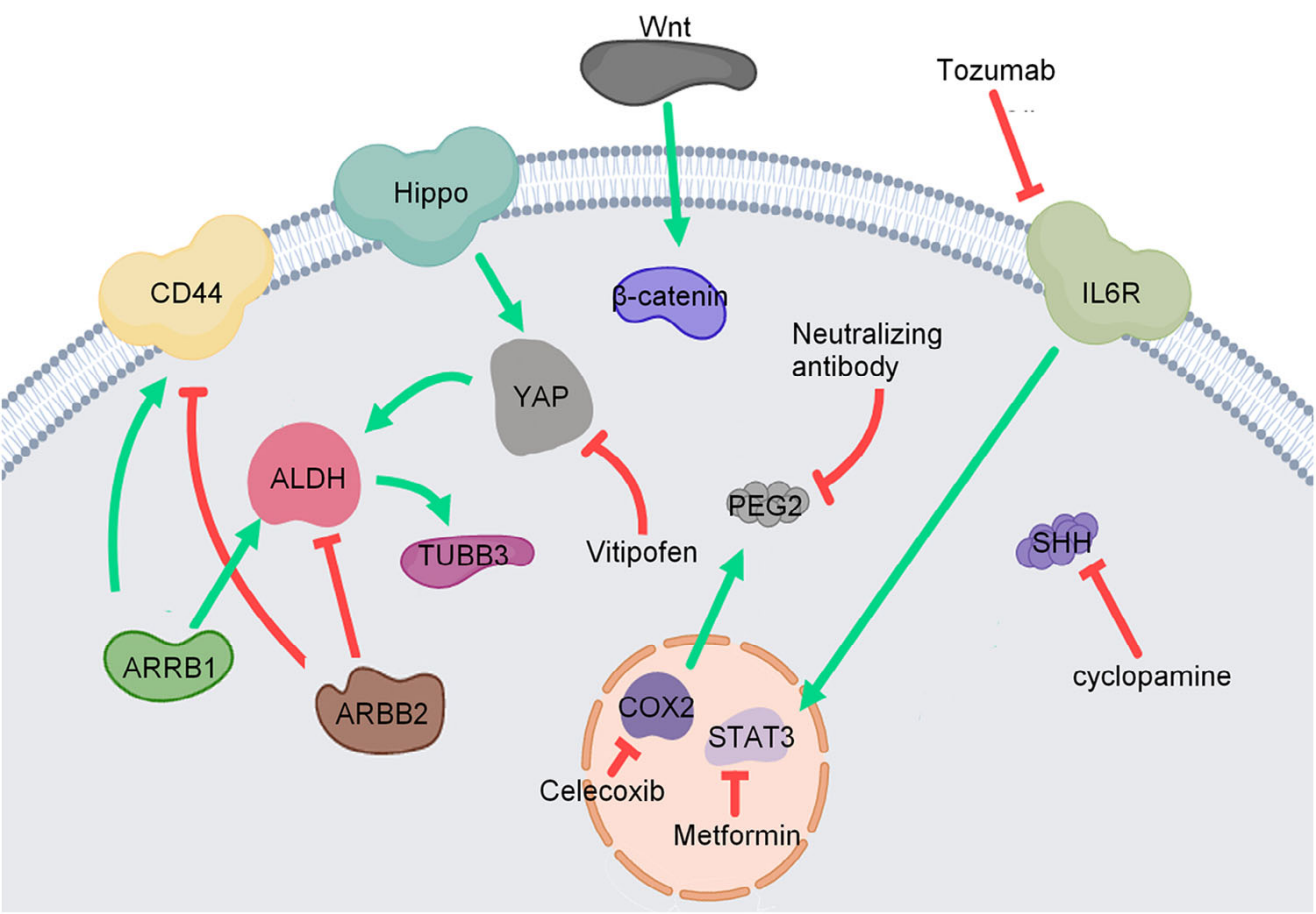
Signaling pathways related to bladder cancer stem cells. It includes Wnt/β‐catenin, Hippo–YAP, IL6/IL6R/STAT3, COX2/PGE2/SOX2, ALDH1A1/TUBB3, ARRB/ALDH/CD44, SHH. Vitipofen inhibits YAP1 to weaken the characteristics of bladder cancer stem cells. Tozumab, a drug targeting IL‐6R, inhibits the effect of IL6/IL6R/STAT3 axis. Metformin can block the progress of bladder cancer by inhibiting stem cell proliferation through the COX2/PGE2/STAT3 axis. PGE2 neutralizing antibody and COX2 inhibitor celecoxib can inhibit the production of PGE2. Cyclopamine can inhibit SHH pathway. ALDH, aldehyde dehydrogenase; ARRB, β‐arrestin; COX2, cyclooxygenase‐2; IL‐6, interleukin 6; IL‐6R, interleukin 6 receptor; PGE2, prostaglandin E2; PGE2, prostaglandin E2; SHH, sonic hedgehog; STAT3, signal transducer and activator of transcription 3; TUBB3, tubulin beta 3; YAP, Yes‐associated protein.

The signaling pathways related to BCSCs are Wnt/β‐Catenin, Hippo–YAP, IL6/IL6R/STAT3, COX2/PGE2/SOX2, ALDH_1A1_/TUBB3, ARRB/ALDH/CD44, and sonic hedgehog (SHH). In addition, some drugs positively inhibit these signaling pathways (Figure 8).

### 6.1 Wnt/β‐catenin pathway regulating BCSCs and associated targeted therapies

The Wnt/β‐Catenin pathway plays a key role in tumor progression and chemoresistance. Maintenance of CSCs in several tumor types is essential. Compared with paclitaxel‐sensitive cells, paclitaxel‐resistant cells exhibit excessive activation of the Wnt/β‐Catenin pathway and increased CSC‐like characteristics. Taxol sensitivity is reduced after β‐Catenin overexpression or when tumor cells grow into tumor globules rich in the CSC‐like phenotype. Furthermore, downregulating β‐Catenin or XAV939 inhibition can render drug‐resistant cells sensitive to paclitaxel. In addition, some MIBC show abnormal expression of β‐Catenin, which is positively correlated with the expression of the CSC marker, ALDH_1A1_. These results confirm that Wnt/β‐Catenin signal transduction advances paclitaxel resistance in BC cells with CSC‐like characteristics.^223^ Veratrol can be used to treat breast cancer, inhibit the Wnt/β‐Catenin pathway, decrease CSC‐like characteristics, and induce autophagy. The CSC features of ovarian cancer are reduced by the combination of imatinib and platinum. However, the therapeutic effects of Wnt signaling inhibition in BCSCs have not been fully elucidated.^224^

### 6.2 Hippo–YAP pathway regulating BCSCs and associated targeted therapies

The Hippo–YAP pathway is dysregulated during the occurrence and development of cancer.^225^ YAP is a key component of the Hippo pathway. The expression levels of YAP in BCSCs are higher than those in non‐CSCs, and silencing YAP decreases the gene expression of ALDH_1A1_. Thus, YAP regulates the stemness of BCSCs by regulating the gene expression of ALDH_1A1_.^226^ Moreover, YAP1 directly induces the expression of SOX2 by combining with its enhancer region, expanding and maintaining the accumulation of urinary tract epithelial CSCs, and promoting the acquisition of drug resistance.^108^ In addition, YAP maintains the characteristics of CSCs by inducing autophagy,^227^ which is critical for the survival, stemness maintenance, and growth promotion of CSCs. In addition, autophagy promotes CSC invasion and metastasis and induces their dormancy to help CSCs develop resistance to the cytotoxic effects of chemotherapy and radiotherapy, thus improving their survival.^228^ The combination of the YAP1 inhibitor, vitipofen, and the COX2 inhibitor, celecoxib, with chemotherapy weakens the characteristics of bladder CSCs and enhances the chemotherapy response.^224^

### 6.3 IL6/IL6R/STAT3 pathway regulating BCSCs and associated targeted therapies

STAT3 is an important regulator of CSCs. It is involved in epithelial‐mesenchymal transformation‐related (EMT) related pathways, which are presumably the main mechanisms of CSC production, and can promote cancer progression by regulating the features of CSCs. Fractionated irradiation promotes the activation of CSC‐related STAT3 signaling pathways in BC cells, and the surviving cells show enhanced migration, invasion, CSC‐like characteristics, and radio‐resistance. The knock‐down of STAT3 expression or inhibition of STAT3 activity significantly reduces the self‐renewal ability and tumorigenicity of antiradiation BC cells. Therefore, STAT3 may be an effective therapeutic target to inhibit the progression, metastasis, and recurrence of BC.^229^ In addition, cancer cells and CSCs induce IL‐6 secretion in an autocrine manner. IL‐6 activation can further lead to the activation of STAT3 in the cytoplasm, which is related to the CSC‐like characteristics of bladder tumors. Thus, the IL6/IL6R/STAT3 pathway can maintain the CSC‐like characteristics of BCSCs. In addition, tozumab, an US FDA‐approved drug targeting IL‐6R, inhibits the effects of IL6/IL6R/STAT3 signal transduction on the occurrence and progression of BCSCs.^230^

### 6.4 COX2/PGE2/SOX2 pathway regulating BCSCs and associated targeted therapies

COX2 is expressed in BC cells but not in normal urinary tract epithelium. Chemotherapy‐induced apoptotic cells release PGE2 and promote CSC proliferation and tumor regeneration during chemotherapy cycles. The COX2/PGE2 signaling pathway induces let‐7 promoter methylation in BC cells, leading to its downregulation and subsequent SOX2 upregulation. SOX2 is a prominent transcription factor that promotes the pluripotency and self‐renewal of embryonic stem cells and induces pluripotent stem cells. The activation of COX2/PGE2 promotes the acquired resistance of basic BC cells to EGFR inhibitors.^107^ Metformin blocks the progression of BC by inhibiting stem cell proliferation through the COX2/PGE2/STAT3 axis.^231^ PGE2 neutralizing antibody and COX‐2 inhibitor, celecoxib, can inhibit PGE2 production, eliminate the repopulation of CSC during chemotherapy intervals, and effectively reduce the progressive performance of chemotherapy resistance.^108^

### 6.5 ALDH1A1/TUBB3 pathway regulating BCSCs and associated targeted therapies

Aldehyde dehydrogenase (ALDH) is involved in important cellular processes such as aldehyde detoxification and retinoic acid synthesis. ALDH activity is associated with drug resistance, which is a CSC‐like characteristic.^232^ ALDH_1A1_ is highly expressed in BCSCs. ALDH levels are directly proportional to BC cell proliferation and regulate the expression via tubulin β3 (TUBB3) through the RA pathway. ALDH_1A1_ decreases the expression of TUBB3. In addition, the ALDH inhibitors, DSF and DEAB, decrease TUBB3 expression. The clinical survival database shows that TUBB3 expression may be linked to poor prognosis in patients with BC.^233^ To date, no specific antagonists have been developed, that specifically inhibit different ALDH isozymes.^234^ Retinoid signal transduction is involved in the regulation and regeneration of basal cells of dermal progenitor cells in the urinary tract.^235^ To target ALDH, careful attention should be paid to the implementation strategy, to avoid severe toxicity.^234^

### 6.6 ARRB/ALDH/CD44 pathway regulating BCSCs and associated targeted therapies

ARRB was initially identified as a multifunctional adaptor protein that plays a key role in tumor growth, metastasis, inflammation, and immunity.^236^ ARRBs regulate stem cell markers and BC characteristics. ARRB1 and ARRB2 possess contradictory functions in regulating the stem cell characteristics of BC. ARRB1 acts as an oncogene and positively regulates the expression of stem cell markers, whereas ARRB2 acts as a tumor suppressor and negatively regulates stem cell characteristics. ARRB2 negatively regulates the CSC markers, ALDH and CD44, and negatively regulates BMI1. B‐cell‐specific Moloney murine leukemia virus insertion site 1 (BMI1) plays a vital role in maintaining the self‐renewal of embryonic and mature stem cells. ARRB2 reduction in BC cells can induce the phosphorylation and activation of STAT3. The basic phenotype of CSC is characterized by the expression of CSC markers KRT14 and KRT17. Remarkably, ARRB2 deregulates KRT14 and KRT17 in BC cells, whereas ARRB1 has the opposite effect.^237^ ARRB1 regulates the metabolic preferences of BCSC‐like cells and acts as a molecular switch to promote glycolytic reprogramming by negatively regulating MPC1 and positively regulating GLUT1/glucose uptake. These observations provide a new therapeutic approach for targeting the metabolism of CSCs as BC cells.^238^ However, the direct targeting of ARRB is difficult for drug development. Furthermore, because of the high homology among subtypes, it is difficult to identify a specific target subtype. Interestingly, a small‐molecule, barbadin, can selectively inhibit the interaction between ARRB and the β2‐adaptin subunit of the clathrin adaptor protein AP2 without impeding the formation of receptor/ARRB complexes. Therefore, barbadin is a potential drug target for ARRB.^237^

### 6.7 SHH pathway regulating BCSCs and associated targeted therapies

The SHH pathway has recently become an important component of cancer initiation, progression, and metastasis. Activation of the SHH signaling pathway plays an important role in BC progression due to its renewable features in stem cells. TGF‐β1 activates the SHH pathway and induces BC cell migration, clonogenic formation, and invasiveness by upregulating EMT and BC stemness. There are still differences in understanding the roles of CSCs and how SHH signaling inhibitors can be used for BC therapy. Cyclopamine, a natural compound from the plant *veratrum californicus*, inhibits the SHH pathway, reduces the activation to target, and inhibits BC.^239^

## 7 DISCUSSION

Using modern biochemical and bioinformatic techniques, the complicated pathological features of BC progression have been successfully explored. The elucidation of BC characteristics has substantially accelerated the development of appropriate targeted therapies. Various new targeted drugs, including five ICIs, an oral FGFR inhibitor, and two ADCs, have been successfully approved for treating BC.^13^ Studies on the pathological molecular features of BC have yielded excellent outcomes. Furthermore, the characterization of metabolic reprogramming and BC stem cells will help develop new therapeutic approaches and establish mechanisms to overcome drug resistance.

However, a subdued response to immunotherapy is a major clinical challenge, with only 20−30% of patients exhibiting positive responses. This can be attributed to two factors. First, standardized and reproducible biomarkers for predicting immunotherapeutic responses are yet to be identified. The identification of novel biomarkers is useful for the biological stratification of patients with BC for precision immunotherapy. Second, the molecular landscape and pathophysiology of BC are not fully understood. Theoretically, immunotherapeutic drugs activate autonomic immunity in patients. In vivo, immune responses can be blocked by multiple suppressor signaling pathways, including the presence of immunosuppressive cells and the activation of immune escape signaling pathways. Therefore, a combination of checkpoint therapies and other treatment agents may improve the treatment outcomes of most patients with BC. Interestingly, new immunotherapeutic alternatives such as mRNA vaccines, cytokines, and antifungal immunotherapies are being developed.^240^, ^241^

Owing to features of the specific and targetable antigens, ADCs are particularly effective for treating patients undergoing chemotherapy with local or metastatic advanced UC. These revolutionary drugs have provided hope to patients with mUC. EV and SG have been approved for clinical applications, and many other ADCs are being assessed in both, preclinical and clinical studies. However, although ADCs exhibit less toxicity and greater therapeutic activity, latent side effects, including rash, neutral granulocytopenia, hematotoxicity, and neurological disorders, have been reported in clinical trials.^114^, ^137^ Thus, these drawbacks must be overcome to develop next‐generation drug candidates. Early evidence suggests that resistance to ADCs involves different signaling pathways, including the loss of payload efficiency, alteration of ADCs processing and internalization, and blockade of antibody attachment.^119^ Therefore, ADCs designs should consider these factors, including lowering the off‐target toxicity of the payload. Studies should aim to investigate the significance of treating various cancers in the early stages, establish drug combinations for better efficacy, overcome drug resistance, and conduct preclinical and clinical trials on the therapeutic potential of ADCs.

Similar to ADCs, small‐molecule targeted drugs have good targeting and achieve high antitumor efficiency and low toxicity. In contrast to ADCs, small‐molecule targeted drugs lack artificial intervention and design and are explored based on a natural signaling pathway or physiological response. TKIs are small‐molecule‐targeted drugs that are commonly used in clinics and have good developmental prospects. Currently, potential therapeutic targets include EGFR, VEGFR, FGFR, and Src in, both, the developed and developing phases.

TKI therapy to achieve breakthrough treatment effect, need rich and reliable preliminary research, need to the pathogenesis of BC and disease characteristics have enough deep grasp, at present, TKI therapy mainly focus on the classic targets and signal pathways, for the exploration of new targets is attractive, but the risk is higher, the current results lack of certain breakthrough.

To enhance the outcomes of TKI therapy for BC, new targets should be identified through active exploration of the novel mechanisms of traditional therapeutic targets. In addition, considering that TKI treatment is prone to the development of drug resistance, there is a need to investigate the associated mechanisms in BC and assess the efficacy of combination treatment strategies. Natural small‐molecule drugs have great potential to overcome drug resistance by inhibiting tyrosine kinases. Their efficacy and safety should also be investigated.

In contrast to immunotherapy, ADCs, and TKI approaches, metabolic therapy for treating BC is still in its early stages and has not yet been clinically applied. There are few drugs, such as metformin, that target metabolism in clinical trials for BC. Although many clinical trials are in progress, the clinical effects have not yet been determined. Preclinical data show that the concentration of metformin required for its antitumor effect is high. Hence, our laboratory has developed a new metformin dosage form, to improve the local metformin concentration in the bladder tumor by perfusion and enhance its anticancer effect. The project is currently undergoing clinical trials (ChiCTR2000038414), and four patients have received treatment with excellent safety and improved efficacy (data not published yet). This clinical trial will continue at multiple clinical sites and with more patients to confirm the clinical benefits. With advances in high‐throughput screening technologies, including transcriptomics, metabolomics, and proteomics, screening for metabolic targets in BC is currently the research focus. Metabolic pathways such as glycolysis, glycogen metabolism, and lipid metabolism are closely associated, and combination therapies targeting different pathways are promising treatments for BC. Thus, in the field of metabolic therapy for BC, further research on the mechanisms of metabolic disorders with specific targets and sufficient clinical evidence is critical for research designs.

Finally, targeting bladder CSCs presents challenges for the treatment of BC. Most bladder CSCs have been found in highly metastatic MIBC but not in NMIBC. Much of the drug resistance induced by chemotherapy or radiotherapy is due to the presence of BCSCs.^228^ Thus, we should first determine the presence of specific markers of BSCSs and then develop treatments with the corresponding targeted drugs. However, the development of stem cell targeting remains challenging. Current research has identified many potential targets; some drugs have been proven effective in preclinical studies, but no clinical trials have been conducted.^223^, ^224^, ^234^, ^239^ Therefore, further research is required to accelerate clinical trials and to provide benefits to patients. In addition, bladder CSCs may emerge in continuous tumors via unknown dynamic mechanisms. Further research on the signaling pathways in BCSCs is required.

## AUTHOR CONTRIBUTIONS

Mei Peng, Xuetong Chu, Yan Peng, Duo Li, and Zhirong Zhang reviewed the literature and drafted the manuscript. Wang and Zhou reviewed relevant literature. Di Xiao and Xiaoping Yang designed and reviewed the manuscript. All the authors have read and approved the final version of the manuscript.

## CONFLICTS OF INTEREST STATEMENT

The authors declare no competing interests.

## ETHICS STATEMENT

Not applicable.

## Data Availability

Not applicable.
